# Host susceptibility and structural and immunological insight of S proteins of two SARS-CoV-2 closely related bat coronaviruses

**DOI:** 10.1038/s41421-023-00581-9

**Published:** 2023-07-28

**Authors:** Xiuyuan Ou, Ge Xu, Pei Li, Yan Liu, Fuwen Zan, Pan Liu, Jiaxin Hu, Xing Lu, Siwen Dong, Yao Zhou, Zhixia Mu, Zhiqiang Wu, Jianwei Wang, Qi Jin, Pinghuang Liu, Jian Lu, Xiangxi Wang, Zhaohui Qian

**Affiliations:** 1 Key Laboratory of Pathogen Infection Prevention and Control (Peking Union Medical College), Ministry of Education, Beijing, China; 2 NHC Key Laboratory of Systems Biology of Pathogens, Institute of Pathogen Biology, Chinese Academy of Medical Sciences & Peking Union Medical College, Beijing, China; 3 State Key Laboratory of Respiratory Health and Multimorbidity, Chinese Academy of Medical Sciences & Peking Union Medical College, Beijing, China; 4 CAS Key Laboratory of Infection and Immunity, National Laboratory of Macromolecules, Institute of Biophysics, Chinese Academy of Sciences, Beijing, China; 5 Key Laboratory of Animal Epidemiology of the Ministry of Agriculture, College of Veterinary Medicine, China Agricultural University, Beijing, China; 6 College of Life Sciences, Peking University, Beijing, China

**Keywords:** Cryoelectron microscopy, Mechanisms of disease

## Abstract

The bat coronaviruses (CoV) BANAL-20-52 and BANAL-20-236 are two newly identified severe acute respiratory syndrome coronavirus 2 (SARS-CoV-2) closely related coronaviruses (SC2r-CoV) and the genome of BANAL-20-52 shares the highest homology with SARS-CoV-2. However, the risk of their potential zoonotic transmission has not been fully evaluated. Here, we determined their potential host susceptibility among 13 different bat species and 26 different animal species, and found that both might have extensive host ranges, indicating high zoonotic transmission potential. We also determined the cryo-EM structures of BANAL-20-52 and BANAL-20-236 S proteins at pH 5.5 and the complex of BANAL-20-236 S1 and *Rhinolophus affinis* ACE2, and found that both trimeric S proteins adopt all three receptor binding domains (RBDs) in “closed” conformation and are more compact than SARS-CoV-2. Strikingly, the unique sugar moiety at N370 of bat SC2r-CoVs acts like a “bolt” and crosses over two neighboring subunits, facilitating the S proteins in the locked conformation and underpinning the architecture stability. Removal of the glycosylation at N370 by a T372A substitution substantially enhances virus infectivity but becomes highly sensitive to trypsin digestion at pH 5.5, a condition roughly mimicking the insectivorous bat’s stomach digestion. In contrast, WT S proteins of SC2r-CoVs showed considerable resistance to trypsin digestion at pH 5.5, indicating that the highly conserved T372 in bat CoVs might result from the selective advantages in stability during the fecal-oral transmission over A372. Moreover, the results of cross-immunogenicity among S proteins of SARS-CoV-2, BANAL-20-52, and BANAL-20-236 showed that A372 pseudoviruses are more sensitive to anti-S sera than T372, indicating that immune evasion might also play a role in the natural selection of T372 over A372 during evolution. Finally, residues 493 and 498 of the S protein affect host susceptibility, and residue 498 also influences the immunogenicity of the S protein. Together, our findings aid a better understanding of the molecular basis of CoV entry, selective evolution, and immunogenicity and highlight the importance of surveillance of susceptible hosts of these viruses to prevent potential outbreaks.

## Introduction

It has been more than three years since the outbreak of the global pandemic of coronavirus disease 2019 (COVID-19). As of April 6^th^, 2023, there are more than 762 million confirmed COVID-19 cases globally, resulting in more than 6.89 million deaths^1^. The COVID-19 pandemic is caused by severe acute respiratory syndrome coronavirus 2 (SARS-CoV-2)^2^, a member of the *Sarbecovirus* of the beta-CoV genus^3^. Several studies, including retrospective analyses of old samples and new surveys of bats in Southeast Asia, Japan, and south China, have led to identification of several new SARS-CoV-2-related coronaviruses (SC2r-CoVs) from various bat species^4^, including RaTG13 from *R. affinis* bat^5^, RshSTT182 and RshSTT200 from *R. shameli* bat^6^, RacCS203 from *R. acuminatus* bat^7^, Rc-o319 in *R. cornutus* bat^8^, RmYN02 from *R. malayanus* bat^9^, and RpYN06 from *R. pusillus* bat^10^, etc, indicating that SARS-CoV-2 may be originated from bats. SC2r-CoVs have also been found in pangolins^11,12^, suggesting that pangolins might play roles in the emergence of SARS-CoV-2. However, the direct zoonotic origin of SARS-CoV-2 remains unknown.

**Fig. 1:**
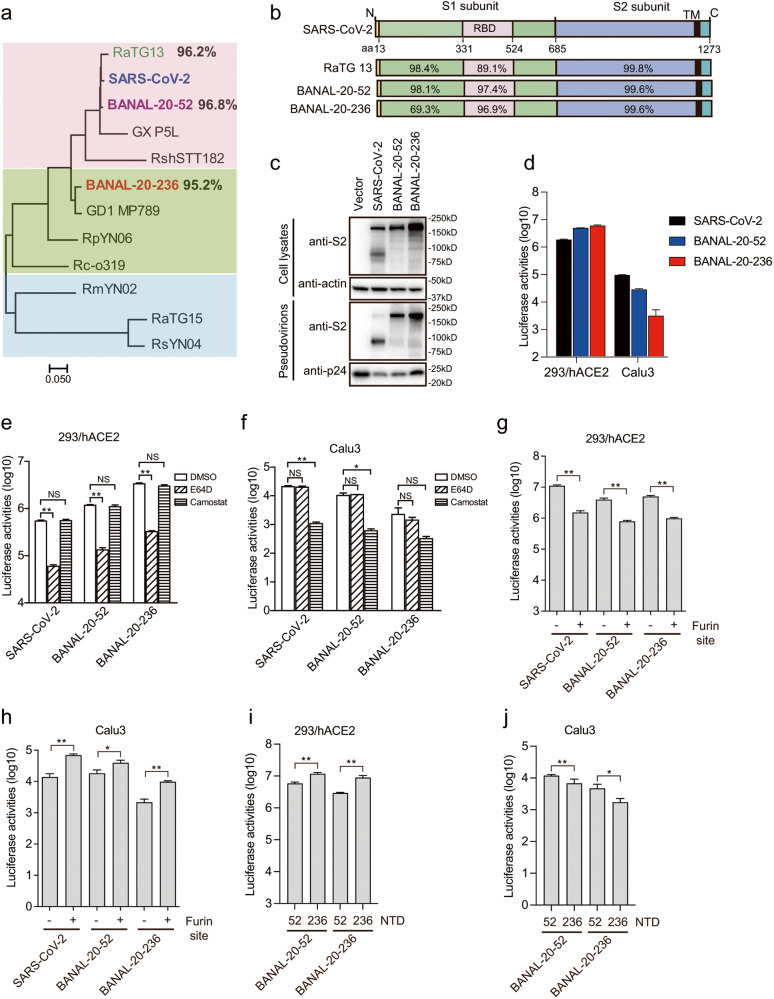
Characterization of S proteins of BANAL-20-52 and BANAL-20-236 on virus entry. **a** Phylogenetic tree of the S proteins of SARS-CoV-2-related CoVs. The maximum-likelihood tree was produced using MEGAX software, based on the alignment of amino acid sequences of S proteins. **b** Schematic diagram of S proteins of the indicated CoVs and the amino acid sequence identities of each region are shown. RBD receptor binding domain, TM transmembrane domain. **c** Western blotting analysis of the S proteins of SARS-CoV-2, BANAL-20-52, and BANAL-20-236 in cells lysates and pseudovirions using rabbit polyclonal anti-SARS-CoV-2 S2 antibody 40590-T62. β-actin and gag-p24 served as loading controls. **d** Entry of SARS-CoV-2 S, BANAL-20-52 S and BANAL-20-236 S pseudovirions on indicated cell lines. 293/hACE2 cells, 293 cells stably expressing human ACE2. **e**, **f** Inhibition of entry of SARS-CoV-2 S, BANAL-20-52 S, and BANAL-20-236 S pseudovirions into 293/hACE2 (**e**) and CaLu3 (**f**) cells by a broad-spectrum cathepsin inhibitor, E64D, or a serine protease inhibitor, camostat. **g**, **h** Transduction of 293/hACE2 (**g**) and CaLu3 (**h**) cells by SARS-CoV-2, BANAL-20-52, and BANAL-20-52 S proteins with or without a furin cleavage site pseudotyped lentiviral particles. **i**, **j** Transduction of 293/hACE2 (**i**) and CaLu3 (**j**) cells by chimeric BANAL-20-52 and BANAL-20-236 S pseudovirions. Data are represented as means ± SD from at least triplicates. *P*-values in **e**–**j** are calculated by unpaired two-sided Student’s *t*-test. *P < 0.05; **P < 0.01; ns, *P* > 0.05.

Recently, a new study in Laos found several new SC2r-CoVs^13^, including BANAL-20-52 from *R. malayanus* bat, BANAL-20-103 from *R. pusillus* bat, and BANAL-20-236 from *R. marshalli* bat, etc. BANAL-20-52 shares about 96.8% nucleotide sequence identity with SARS-CoV-2, the highest among all known SC2r-CoVs. Strikingly, its spike (S) protein, one of the least conserved parts of CoV genomes, shares over 98% of amino acid sequence identity with SARS-CoV-2 (Fig. 1a, b), with N-terminal domain (NTD) at 98.1% and receptor binding domain (RBD) at 97.4%. In contrast, both the genomes of BANAL-20-103 and BANAL-20-236 share about 95.2% nucleotide sequence identity with SARS-CoV-2 and 98.4% nucleotide sequence identity with each other. The S proteins of BANAL-20-103 and BANAL-20-236 are also highly similar, and there are only five amino acids difference between them, one in NTD, one in RBD, one close to the junction site between S1 and S2 subunits, and two in S2 subunit. The S proteins of BANAL-20-103 and BANAL-20-236 share about 90.4% amino acid sequence identity with SARS-CoV-2. While their NTDs are only about 69.3% identical to that of SARS-CoV-2, their RBDs share nearly 97% similarity with SARS-CoV-2 (Fig. 1b). Of note, the RBD of BANAL-20-103 is identical to that of BANAL-20-52, and all S proteins of BANAL CoVs lack the furin cleavage site between S1 and S2. BANAL CoVs also use human angiotensin-converting enzyme 2 (hACE2) as the receptor^13^.

Many animals are susceptible to SARS-CoV-2 infection. We and others recently also showed that bat SC2r-CoVs like RaTG13 could utilize various animal ACE2s for virus entry^14,15^, indicating the potential broad host ranges of bat SC2r-CoVs. In this study, we characterized the entry pathway mediated by BANAL-20-52 and BANAL-20-236 S proteins, determined the potential host susceptibility of BANAL-20-52 and BANAL-20-236 among various bat and animal species, solved cryo-EM structures of S proteins of BANAL-20-52 and BANAL-20-236, and also evaluated their immunogenicity and cross-neutralization activities among S proteins of BANAL-20-52, BANAL-20-236, and SARS-CoV-2.

## Results

### Characterization of viral entry mediated by BANAL-20-52 and BANAL-20-236 S protein

Since complete S protein sequences of BANAL-20-103 and BANAL-20-236 are highly similar and RBDs of BANAL-20-103 and BANAL-20-52 are identical, we selected BANAL-20-52 and BANAL-20-236 S proteins in this study. The FLAG-tagged S protein of BANAL-20-52 or BANAL-20-236 were expressed well in Human embryonic kidney (HEK) 293 T cells (Fig. 1c; Supplementary Fig. S1) and incorporated into pseudovirions as efficiently as SARS-CoV-2 S proteins (Fig. 1c; Supplementary Fig. S1). Of note, only full-length S proteins (~180–200 kDa) of BANAL-20-52 and BANAL-20-236 were detected in cell lysate and pseudovirions, consistent with lack of the furin site between S1 and S2 subunits. However, both bat S proteins highly efficiently mediated cell–cell fusion at a level similar to SARS-CoV-2 S protein (Supplementary Fig. S2), when the S-expressing cells were mixed with HEK293 cells stably expressing hACE2 (293/hACE2) in the presence of exogenous trypsin, indicating that fusion potential of both S proteins could be primed and activated by trypsin cleavage and hACE2 binding.

HEK293/hACE2 and human lung cancer cells, CaLu3, were used to evaluate the transduction efficiency of BANAL-20-52 and BANAL-20-236 S pseudovirions (Fig. 1d). BANAL-20-52 and BANAL-20-236 S pseudoviruses entered 293/hACE2 cells very efficiently, even slightly better than SARS-CoV-2 (WH strain), in agreement with the higher affinity of hACE2 with RBDs of BANAL-20-52 and BANAL-20-236 than SARS-CoV-2^13^. Similar to SARS-CoV-2^16,17^, bafilomycin A1 (BFA), apilimod, and E64D but not camostat significantly reduced entry of BANAL-20-52 and BANAL-20-236 S pseudovirions into 293/hACE2 cells (Fig. 1e; Supplementary Fig. S3), indicating that endocytosis might also be the major entry pathway on 293/hACE2 cells by BANAL-20-52 and BANAL-20-236 S pseudoviruses and PIKfyve and cathepsins might be important. In contrast, entry of CaLu3 cells by BANAL-20-52 and BANAL-20-236 S pseudovirions was markedly inhibited by camostat but not BFA, apilimod, or E64D (Fig. 1f; Supplementary Fig. S3), indicating that BANAL-20-52 and BANAL-20-236 might enter CaLu3 cells through the cell surface and serine protease might be critical. However, compared to SARS-CoV-2 S, BANAL-20-52 and BANAL-20-236 S pseudovirions transduced CaLu3 cells less efficiently (Fig. 1f). The furin site between S1 and S2 enhances the serine protease-mediated S protein activation and entry on CaLu3 cells^18,19^. An exogenous furin site was inserted between S1 and S2 of BANAL-20-52 and BANAL-20-236 S proteins and confirmed by western blot analysis showing a significant increase of cleaved S protein incorporation into pseudovirions, compared to WT BANAL-20-52 and BANAL-20-236 S proteins (Supplementary Fig. S4). While the presence of a furin site might reduce S protein stability and decrease the transduction efficiency of both BANAL-20-52 and BANAL-20-236 S pseudovirions on 293/hACE2 cells by about 5-fold (Fig. 1g), the addition of furin site markedly increased the entry of both BANAL-20-52 and BANAL-20-236 S pseudovirions on CaLu3 cells by ~2- and 5-fold, respectively (Fig. 1h). Of note, BANAL-20-236 S showed markedly lower transduction efficiency on CaLu3 cells than BANAL-20-52 (Fig. 1h), regardless of whether the furin site is present. Since their RBD sequences share a high similarity, we reasoned that the sequence differences in their NTDs might contribute to this discrepancy. The NTDs of BANAL-20-52 and BANAL-20-236 were swapped, and they had almost no effect on S protein expression and incorporation into pseudovirions (Supplementary Fig. S5). The transduction on 293/hACE2 and CaLu3 cells was evaluated. While the replacement of BANAL-20-52 NTD with BANAL-20-236 NTD in BANAL-20-52 S protein increased the transduction on 293/hACE2 cells by about 2-fold (Fig. 1i), its transduction on CaLu3 cells was reduced by more than 40% (Fig. 1j). In contrast, the substitution of BANAL-20-236 NTD with BANAL-20-52 NTD in BANAL-20-236 S protein decreased the infection on 293/hACE2 cells by 3-fold (Fig. 1i), while increased the transduction on CaLu3 cells by more than 2.7-fold (Fig. 1j). These results suggest that NTD might play an important role in the entry of CaLu3 cells.

### Cryo-EM structures of BANAL-20-52 and BANAL-20-236 S proteins reveal a compact architecture for the proteins

**Fig. 2:**
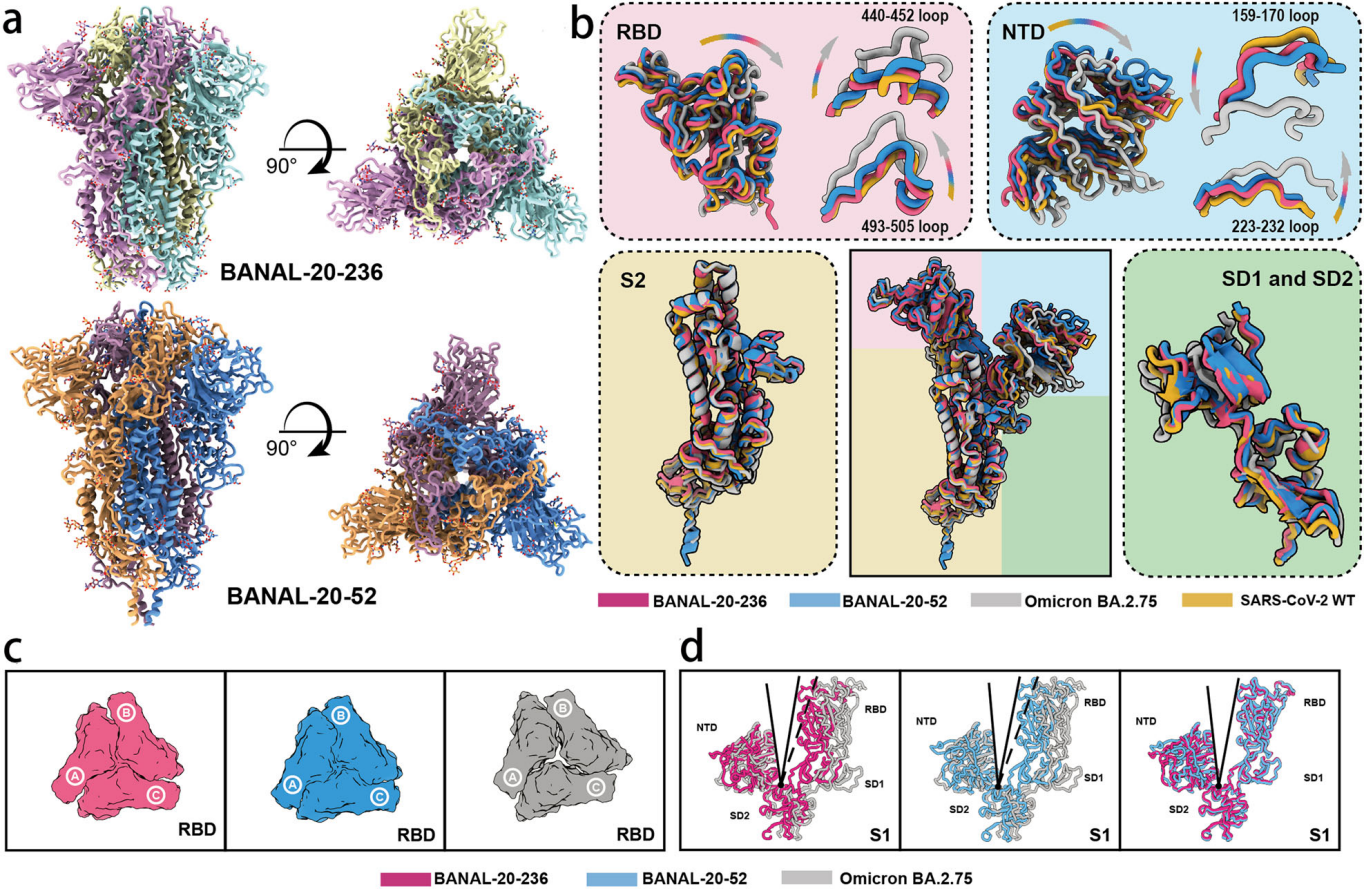
Cryo-EM structures of the S ectodomain trimers of BANAL-20-52 and BANAL-20-236. **a** Cryo-EM map of BANAL-20-52 (PDB ID: 8HXJ) and BANAL-20-236 (PDB ID: 8I3W) S trimer. Three protomers of BANAL-20-236 were colored in yellow, cyan, and magenta, and three protomers of BANAL-20-52 were colored in blue, orange, and pink. Left, side view; right, top view. **b** The monomeric structures of BANAL-20-236 (magenta) (PDB ID: 8I3W) and BANAL-20-52 (blue) (PDB ID: 8HXJ) S proteins are superimposed to those of SARS-CoV-2 WH strain (PDB ID: 7XU6) (yellow) and Omicron BA.2.75 (PDB ID: 7YQW) (gray). The superimposed individual domain structures and the indicated loops (RBD (top left), NTD (top right), S2 (bottom left) and SD1 and SD2 (bottom right)) are also shown. Both SARS-CoV-2 and omicron BA.2.75 S proteins here are in “down” conformations at an acidic pH. **c** Top view: Comparison of inter-RBD contacts of the BANAL-20-236 (red), BANAL-20-52 (blue) and Omicron BA.2.75 (gray) S-trimers. **d** Structural comparison of the angle formed by NTD-SD2-RBD of BANAL-20-236/52 and Omicron BA.2.75. Red, BANAL-20-236; blue, BANAL-20-52; gray, omicron BA.2.75.

To gain structural insight of S proteins of BANAL-20-52 and BANAL-20-236, we determined the cryo-EM structures of these two S proteins and compared them with those of SARS-CoV-2. The trimeric ectodomains of BANAL-20-52 and BANAL-20-236 S proteins with foldon^20,21^ at their C-terminus were stabilized with 2P mutations at prefusion conformation^22^, expressed in Expi293F cells, and purified by affinity chromatography and size-exclusion chromatography. Initial preparations at pH 8.0 were not suitable for their structure determination. Subsequently, the preparation pH was changed to 5.5, and data were successfully collected and analyzed. The resulting cryo-EM micrographs at pH 5.5 yielded one trimeric cryo-EM structure with C3 symmetry for each bat CoV S protein, with a resolution of 3.52 Å for BANAL-20-52 and 2.85 Å for BANAL-20-236 (Fig. 2; Supplementary Fig. S6). Of note, only one stable prefusion conformation at all “down” was found for both BANAL-20-236 and BANAL-20-52 trimeric S proteins (Fig. 2a). In comparison, the trimeric S proteins of various SARS-CoV-2 variants, including Alpha, Beta, Kappa, and Omicron, generally have two or more prefusion conformations^23–25^. The structures of the BANAL-20-236 and BANAL-20-52 S proteins highly resemble each other (Fig. 2) and all glycans on these two trimers are almost structurally identical to those on SARS-CoV-2 variants yielded from mammalian expression system. In line with most coronaviruses, the regions (residues 677–685 of BANAL-20-52 and residues 673–681of BANAL-20-236) corresponding to the furin site of SARS-CoV-2 were disordered (Supplementary Fig. S7), indicating the high structural flexibility. Superimposition of monomeric structures of BANAL-20-52 and BANAL-20-236 S proteins over those of WT and BA.2.75 solved at pH 5.5 reveals a characteristic overall architecture with slight conformational shifts in domain arrangements, in particular apical RBD and NTD (Fig. 2b). Structural comparisons place BANAL-20-52 and BANAL-20-236 between WT and Omicron, which is largely consistent with functional observations that BANAL-20-52 and BANAL-20-236 can enter into host cells through endocytosis as well as on the cell surface. Unexpectedly, BANAL-20-52 and BANAL-20-236 S-trimers exhibit a more compact upper architecture (Fig. 2c), which primarily arises from tight packing between RBD and NTD within one monomer as well as three RBD clustering (Fig. 2d). Apart from protein–protein interactions within the monomer, each NTD is also contacted by its two adjacent monomers with 34 and 31 inter-subunit contacts of residue pairs in BANAL-20-52 and BANAL-20-236, respectively (Supplementary Fig. S8 and Tables S1 and S2). Due to high diversity of animo acid sequences in NTDs between BANAL-20-52 and BANAL-20-236, ~22% of inter-subunit contacts mediated by NTD differs each other. In-silico analysis of the NTD swap revealed that the overall numbers of contacts between the NTDs and its neighboring subunits for BANAL-20-236 S with 52 NTD and BANAL-20-52 S with 236 NTD are 37 and 30, respectively (Supplementary Table S3), and the number of NTD-mediated inter-subunit contacts appears to correlate transduction on 293/hACE2 cells (Fig. 1i) but not on CaLu3 (Fig. 1j). However, the exact molecular mechanism on how NTD modulates viral entry pathway needs to be further explored.

### Susceptibility of bat ACE2s by BANAL-20-52 and BANAL-20-236 S pseudoviruses

**Fig. 3:**
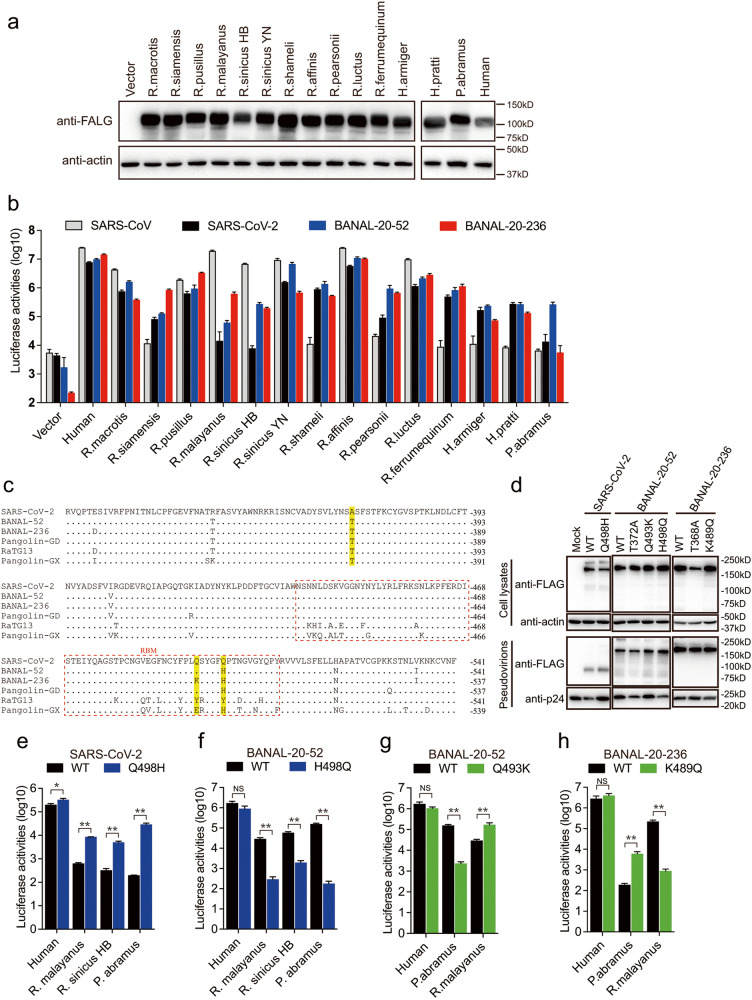
Susceptibility of different bat ACE2s by BANAL-20-52 S and BANAL-20-236 S pseudovirions. **a** Western blotting analysis of cell lysates of HEK293 cells transiently over-expressing FLAG-tagged ACE2 orthologs from different bat species using anti-FLAG M2 antibodies. β-actin served as the loading controls. **b** Transduction of HEK293 cells expressing ACE2 orthologs from different bat species by SARS-CoV S, SARS-CoV-2 S, BANAL-20-52 S, and BANAL-20-236 S pseudovirions. The experiments were done in triplicate and repeated at least three times. One representative is shown with error bars indicating SD. **c** Alignment of RBD amino acid sequences from SARS-CoV-2-related sarbecoviruses. RBMs, receptor binding motifs, are indicated by dashed red line box. Residues 372, 493, and 498 are shaded in yellow background. **d** Detection of SARS-CoV-2 S, BANAL-20-52, or BANAL-20-236 S mutants in cell lysates and pseudovirions by western blot assay using anti-FLAG M2 antibodies. β-actin and gag-p24 served as loading controls (cell lysates, top panel; pseudovirions, bottom panel). **e**, **f** Transduction of 293 cells expressing human ACE2, *R. malayanus* ACE2, *R. sinicus* HB ACE2, or *P. abramus* ACE2 by WT and Q498H mutant SARS-CoV-2 S pseudovirions (**e**), and WT and H498Q mutant BANAL-20-52 S pseudovirions (**f**). **g**, **h** Transduction of 293 cells expressing human ACE2, *R. malayanus* ACE2, or *P. abramus* ACE2 by WT and Q493K mutant BANAL-20-52 S pseudovirions (**g**), and WT and K489Q mutant BANAL-20-236 S pseudovirions (**h**). Data are represented as means ± SD from at least triplicates. *P*-values in **e**–**h** are calculated by unpaired two-sided Student’s *t*-test. *P < 0.05; **P < 0.01; ns, *P* > 0.05.

Although BANAL-20-52, BANAL-20-103, and BANAL-20-236 were first detected in *R. malayanus*, *R. pusillus*, and *R. marshalli* bats^13^, respectively, other bat species might also be susceptible to their infection. We then investigated whether 14 ACE2s from 13 different bat species (Supplementary Table S4), including *R. malayanus* and *R. pusillus* bats, could mediate the entry of BANAL-20-52 and BANAL-20-236 S pseudovirions. Unfortunately, we could not find the sequence information of ACE2 of the *R. marshalli* bat. All 13 bat species are known to harbor sarbecoviruses^5–10,13,26–28^, and there are 10 *Rhinolophus* bats, 2 *Hipposideros* bats, and 1 *Pipistrellus* bat. Two different *R. sinicus* bat ACE2s (one from Yunnan, *R. sinicus* YN, and the other from Hubei, *R. sinicus* HB) were also included. All FLAG-tagged bat ACE2 proteins were expressed (Fig. 3a) and present on the cell surface (Supplementary Fig. S9) at levels similar to or even higher than hACE2. Next, HEK293 cells expressing different bat ACE2s were transduced with either BANAL-20-52 or BANAL-20-236 S pseudovirions. We also included SARS-CoV and SARS-CoV-2 S pseudovirions for comparison. To our surprise, among fourteen different bat ACE2s, only seven bat ACE2s, including two *R. sinicus* ACE2s, were susceptible to transduction by SARS-CoV S pseudovirions; all belong to *rhinolophus* bats (Fig. 3b). There were seven bat ACE2s highly susceptible to SARS-CoV-2 S pseudovirus transduction with an increase in luciferase activities by more than 100-fold over vector control and four additional bat ACE2s (*R. siamensis, R. pearsonii, H. armiger*, and *H. pratti*) showed a modest increase in luciferase activities, ranging from 18.6-fold to 62.2-fold (Fig. 3b). Two *R. sinicus* ACE2s showed significantly different susceptibility to SARS-CoV-2 infection, in agreement with our previous report^14^. In contrast, BANAL-20-52 and BANAL-20-236 S pseudovirions showed broader susceptibility among bat ACE2s. All fourteen bat ACE2s were susceptible to transduction by BANAL-20-52 and BANAL-20-236 S pseudovirions (Fig. 3b), supporting the bat origin of these two CoVs. When transduced by BANAL-20-52 S pseudovirions, twelve bat ACE2s showed more than a 100-fold increase in transduction over vector control, and the remaining two bat ACE2s, *R. siamensis* and *R. malayanus*, showed 74.7- and 35.8-fold increase over vector control, respectively. Thirteen out of fourteen bat ACE2s (except for *P. abramus*) showed more than a 300-fold increase in luciferase activities over mock control when transduced with BANAL-20-236 (Fig. 3b). These results highlight the potential broad host range of these two SC2r-CoVs among bats, indicating that BANAL-20-52 and BANAL-20-236 might have well adapted in bat population. Of note, only five bat ACE2s (*R. macrotis*, *R. pusillus*, *R. sinicus* YN, *R. affinis*, and *R. luctus*) are susceptible to transduction by all four CoV S pseudovirions, and all of them are *rhinolophus* bats.

Despite very high sequence identity among RBDs of SARS-CoV-2, BANAL-20-52, and BANAL-20-236, their S pseudovirions showed different levels of cell entry efficiency using ACE2s of *R. malayanus, R. sinicus* HB, and *P. abramus* bats. The residues in the receptor binding motif (RBM) (Fig. 3c) are responsible for the recognition of ACE2, and there are only two residues, 493 and 498 (SARS-CoV-2 numbering), in RBMs different among SARS-CoV-2, BANAL-20-52, and BANAL-20-236. Since RBMs of SARS-CoV-2 and BANAL-20-52 differ only in residue 498, Q498H and H498Q substitutions were introduced into SARS-CoV-2 and BANAL-20-52 S proteins, respectively. The Q498H and H498Q changes almost had no effect on S protein expression, or incorporation into pseudovirions (Fig. 3d). However, the Q498H mutation in SARS-CoV-2 S significantly increased pseudoviral entry on the ACE2s of *R. malayanus, R. sinicus* HB, and *P. abramus* bats (Fig. 3e), likely resulting from strong hydrophobic interaction between H498 of S protein and H41 or Y41 of ACE2s (Supplementary Fig. S10). In contrast, the H498Q change in BANAL-20-52 S led to a markedly reduced efficiency of virus entry on these three bat ACE2 cells (Fig. 3f), further confirming that residue 498 of S protein might be critical in recognition of these three bat ACE2s, with a preference of H over Q.

BANAL-20-52 and BANAL-20-236 S pseudovirions showed different transduction efficiency on *R. malayanus* and *P. abramus* ACE2s, and two S proteins differ in only one residue, 493 (SARS-CoV-2 and BANAL-20-52 S protein numbering, equivalent to residue 489 in BANAL-20-236 S protein), located in RBM. A Q493K substitution was introduced into BANAL-20-52 S and K489 was replaced with Q (K489Q) in BANAL-20-236 S protein (Fig. 3g). While the Q493K mutation substantially reduced the transduction efficiency on *P. abramus* ACE2, it enhanced infection in cells expressing *R. malayanus* ACE2 by over 5.9-fold (Fig. 3g). In contrast, K489Q mutant BANAL-20-236 S pseudovirions showed a marked increase in entering cells expressing *P. abramus* ACE2 by more than 31.8-fold, whereas it almost abrogated its infectivity on *R. malayanus* ACE2-expressing cells (Fig. 3h). Collectively, these results indicate that residue 493 in BANAL-20-52 S or 489 in BANAL-20-236 S play a crucial role in interacting with *P. abramus* and *R. malayanus* bat ACE2s.

### Residue 372 in S proteins modulates viral infectivity and stability

**Fig. 4:**
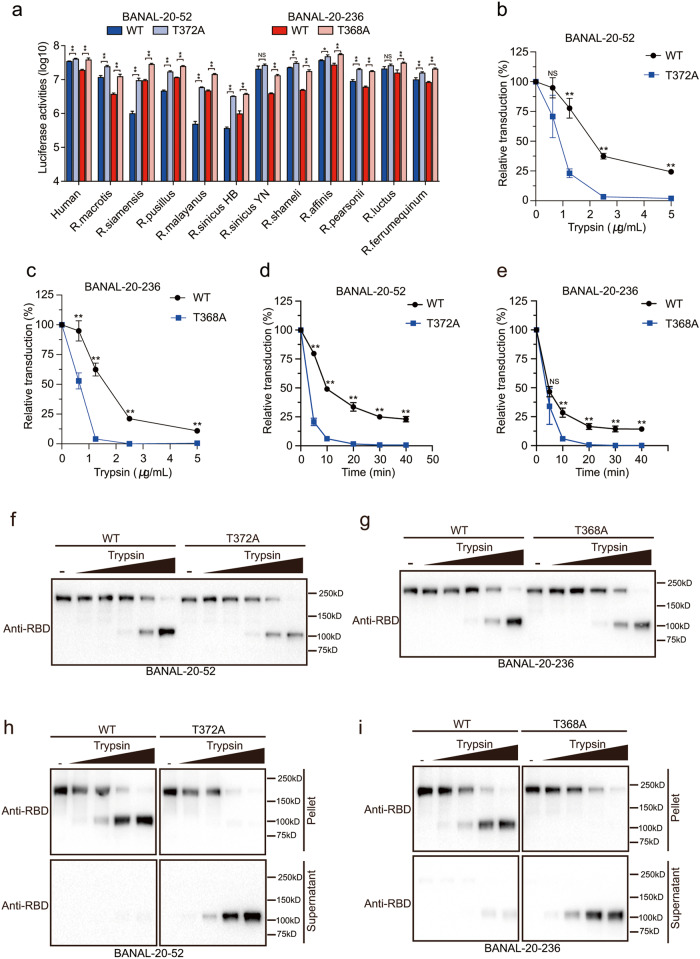
Residue 372 of S proteins modulates viral infectivity and stability. **a** Transduction of 293 cells expressing various bat ACE2s by WT or T372A (or T368A) mutant S pseudovirions. **b**, **c** WT and T372A (or T368A) mutant S pseudovirions, BANAL-20-52 (**b**), BANAL-20-236 (**c**), were pre-treated with indicated concentration of trypsin (0.625, 1.25, 2.5, and 5 μg/mL) at pH 5.5 for 10 min and the remaining infectivity was assayed on 293/hACE2 cells. **d**, **e** WT and T372A (or T368A) mutant S pseudovirions, BANAL-20-52 (**d**), BANAL-20-236 (**e**), were pre-treated with 2.5 μg/mL trypsin for various time points and the remaining infectivity was assayed on 293/hACE2 cells. **f**, **g** Western blot analysis of pseudovirions, BANAL-20-52 (**f**), BANAL-20-236 (**g**), treated with different concentrations of trypsin (0.313, 0.625, 1.25, 2.5, and 5 μg/mL) for 10 min using anti-SARS-CoV-2 RBD antibodies. **h**, **i** Detection of association of cleaved S1 subunits with pseudovirions, BANAL-20-52 (**h**), BANAL-20-236 (**i**), by western blot analysis, after different concentrations of trypsin (0.625, 1.25, 2.5, and 5 μg/mL) treatment at pH 5.5 for 10 min. Trypsin-treated pseudovirions were pelleted by centrifugation to separate the supernatants and pellets, and the cleaved S1 subunits in the supernatants and pellets were separated in a 10% SDS-PAGE and detected using rabbit polyclonal anti-SARS-CoV-2 RBD antibodies. Data are represented as mean ± SD from at least triplicates. *P*-values in **a**–**e** are calculated by unpaired two-sided Student’s *t*-test. *P < 0.05; **P < 0.01; ns, *P* > 0.05.

Previously Kang et al. showed that Ala is favored over Thr at residue 372 in SARS-CoV-2 and A372T substitution reduced virus infectivity by more than 20-fold^29^, possibly through its role in modulating “down” and “up” conformations of RBDs in S proteins^30^. BANAL-20-52 and BANAL-20-236 S proteins have Thr at the corresponding positions (T372 in BANAL-20-52 and T368 in BANAL-20-236). To verify the role of residue 372 in infectivity, we replaced T372 in BANAL-20-52 and T368 in BANAL-20-236 with Ala. While neither the T372A mutation in BANAL-20-52 S nor T368A mutation in BANAL-20-236 had any effect on S protein expression and virion incorporation (Fig. 3d), both mutant S pseudovirions showed a noticeable increase in luciferase activities on cells expressing hACE2 and bat ACEs over WT BANAL-20-52 and BANAL-20-236 S pseudovirions, respectively (Fig. 4a), consistent with the previous reports on SARS-CoV-2 and RaTG13^29,30^. There are at least 38 unique sequences of S proteins of SC2r-CoVs available in Genbank, and the glycosylation sequon (NxT/S) around residue 372 (numbering from SARS-CoV-2 S) is conserved among S proteins of all known SC2r-CoVs (Supplementary Fig. S11). Why do all bat SC2r-CoVs retain T372, not A372, in their spike proteins, even though the A372 mutant showed substantially higher infectivity than T372? Since the fecal-oral route plays a vital role in bat CoV transmission among bats^31,32^, we hypothesized that fecal-oral transmission might favor S proteins in all "down" conformation during natural selection, and T372A change might cause some RBDs to assume “up” conformation, which might be detrimental for the survival of S proteins during their passage through the bat stomach. The pH of an insectivorous bat stomach is around 5.6^33^. To test this hypothesis, WT and T372A mutant S pseudovirions were treated with TPCK trypsin at pH 5.5 at 37 °C, a condition roughly mimicking bat stomach digestion. With increase of trypsin concentration, both WT and T372A pseudovirions lost significant amount of infectivity (Fig. 4b, c). However, the speed and extent of infectivity loss varied significantly between WT and T372A mutants (Fig. 4b, c). While a brief 10 min treatment of trypsin at 2.5 μg/mL resulted in over 96.6% and 99.9% loss of infectivity for BANAL-20-52 T372A and BANAL-20-236 T368A mutants, respectively, WT BANAL-20-52 and BANAL-20-236 S pseudovirions retained more than 37% and 21% of infectivity (Fig. 4b, c). Moreover, even after 40 min digestion with trypsin at 2.5 μg/mL, WT BANAL-20-52 and BANAL-20-236 pseudoviruses still retain over 23% and 14% of infectivity, respectively, whereas T372A and T368A mutants almost completely lost infectivity (Fig. 4d, e).

To further determine how T372A and T368A mutant pseudoviruses become more sensitive to protease digestion at pH 5.5 than WT, trypsin-treated pseudovirions were pelleted by centrifugation and analyzed by western blot assay with polyclonal anti-SARS-CoV-2 RBD antibodies. Regardless of WT and mutant S pseudoviruses, trypsin cleavage of S proteins at pH 5.5 and 37 °C generated a new band, a size roughly corresponding to S1 (Fig. 4f, g). However, the newly generated S1-like fragments from WT pseudovirions were mainly associated with the virions (Fig. 4h, i). In contrast, most of the S1-like fragments from T372A and T368A mutant pseudoviruses were present in supernatant and dissociated from virions (Fig. 4h, i), resulting in loss of infectivity. Similar phenomena were also observed for S proteins of several other SC2r-CoVs, including RaTG13, RaTG15, pangolin-CoV-GD, etc. (Supplementary Fig. S12). These results further support our hypothesis that the T372A and T368A substitutions might cause some RBDs to adopt “up” conformation in S proteins, which might be detrimental for viruses when exposed to proteases in bat stomachs.

**Fig. 5:**
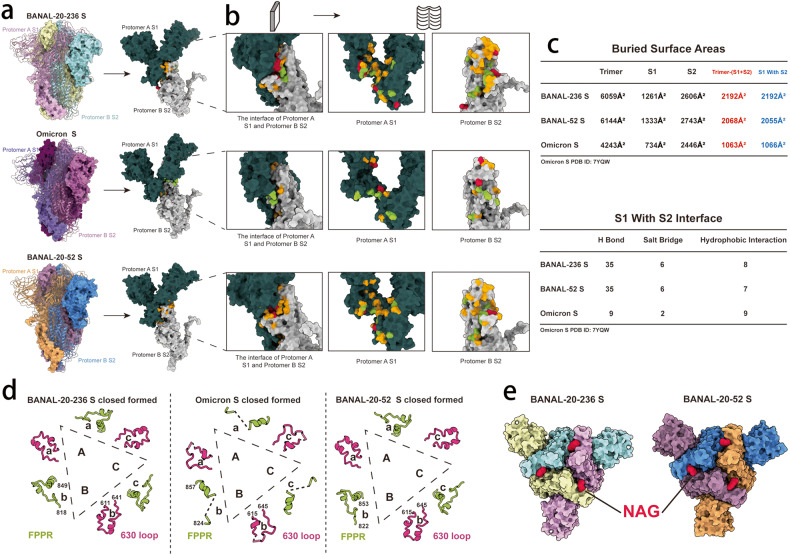
Structural analysis of BANAL-20-236, BANAL-20-52, and SARS-CoV-2 S trimmers. **a** Left: trimeric structures of BANAL-20-236, BANAL-20-52, and omicron BA.2.75S proteins, S1 of protomer A and S2 of protomer B were shown as ribbon, the rest of trimer was shown as surface presentation. Top, BANAL-20-236; middle, omicron BA.2.75; bottom, BANAL-20-52. Right: surface presentation of the S1 of protomer A (dark green) and the S2 of protomer B (gray). Omicron S PDB ID: 7YQW. **b** Magnified open flat book view of interface interaction of S1 of protomer A and S2 of protomer B. The first column: the side view of S1-S2 interface; the second and third columns: Open flat book view of interface interactions of S1 and S2 in the first column. Bright yellow, amino acid residues forming hydrogen bonds; red, amino acid residues involved in salt bridges; green, amino acid residues involved in hydrophobic interactions. **c** Top: the buried surface areas of all “closed” trimeric S proteins of BANAL-20-236, BANAL-20-52 and Omicron BA.2.75 at pH 5.5. Trimer: all buried surface areas; S1: areas of the interaction interface between S1 subunits only; S2: areas of the interaction interface between S2 subunits only; Trimer-(S1 + S2): Trimer column-S1 column-S2 column; S1 with S2: areas of interaction interface between S1 and S2. Bottom: the number of the residues involved in hydrogen bonding, salt bridge, and hydrophobic interaction at the interface of S1 and S2 interaction. Omicron BA.2.75S PDB ID: 7YQW. **d** Structural comparison of the 630 loop (rose) (BANAL-20-236: residues 611–641, BANAL-20-52: residues 615–645, Omicron: residues 615–645) and FPPR (green) (fusion peptide proximal region) (BANAL-20-236: residues 818–849, BANAL-20-52: residues 822–853, Omicron: residues 824–857). Dashed lines indicate the missing gaps. Omicron S PDB ID: 7YQW. **e** Top views of the trimeric S proteins of BANAL-20-236 and BANAL-20-52. The NAG moiety at N370 of S proteins was shown in red.

The notion of the stable T372 S proteins of BANAL-20-52 and BANAL-20-236 is also supported by structural comparison of S proteins of BANAL-20-52/236 with omicron BA.2.75 (Fig. 5a, b). There are thirty-five hydrogen bonds (yellow color) and six salt bridges (red color) formed between S1 and adjacent S2 subunits in both BANAL-20-52 and BANAL-20-236 (Fig. 5b, c). In contrast, there are only nine hydrogen bonds and two salt bridges for BA.2.75, further supporting the more compact and stable structures of BANAL-20-52 and BANAL-20-236 (Fig. 5b, c). We also measured the total interaction areas (Fig. 5c) between monomers within trimeric S proteins, which are 6012 Å^2^ and 6026 Å^2^ for BANAL-20-52 and BANAL-20-236, respectively, and significantly larger than that of BA.2.75 at 4676 Å^2^ (Fig. 5c). The difference mainly results from markedly larger contact made by the S1 subunit of BANAL-20-52 and BANAL-20-236 with adjacent S1 and S2 subunits than BA.2.75 (S1 and S1-S2 in Fig. 5c). The 630 loop and the fusion peptide proximal region (FPPR) play critical roles in the modulation of the S protein fusogenicity through structural rearrangements^34^. Compared to the relatively flexible and partially disordered 630 loop and FPPR in BA.2.75 S protein, the 630 loop and FPPR are well ordered in S proteins of both BANAL-20-52 and BANAL-20-236 (Fig. 5d; Supplementary Fig. S13). The 630 loop inserts into a groove formed between the NTD and SD2 of the same monomer in both bat CoV S proteins, stabilizing the SD2 structure, while the relatively rigid FRRP loop forms a steric hindrance to restrict the movement of SD1 (Supplementary Fig. S14). The 630 loop and the FPPR together help keep the bat CoV S proteins at all “down” conformation. Lastly, there is an additional NAG modification at N370 of S proteins of BANAL-20-52/236, compared to SARS-CoV-2 S protein. The NAG370 locates at two neighboring RBD interface and forms hydrogen bonds with Q493 in BANAL-20-52 and with K489 in BANAL-20-236, respectively (Fig. 5e; Supplementary Fig. S15), acting like a “bolt” to keep S protein in all “down” conformation at pH 5.5 (Fig. 5e).

### Cryo-EM structure of RaACE2/BANAL-20-236 S1 complex

**Fig. 6:**
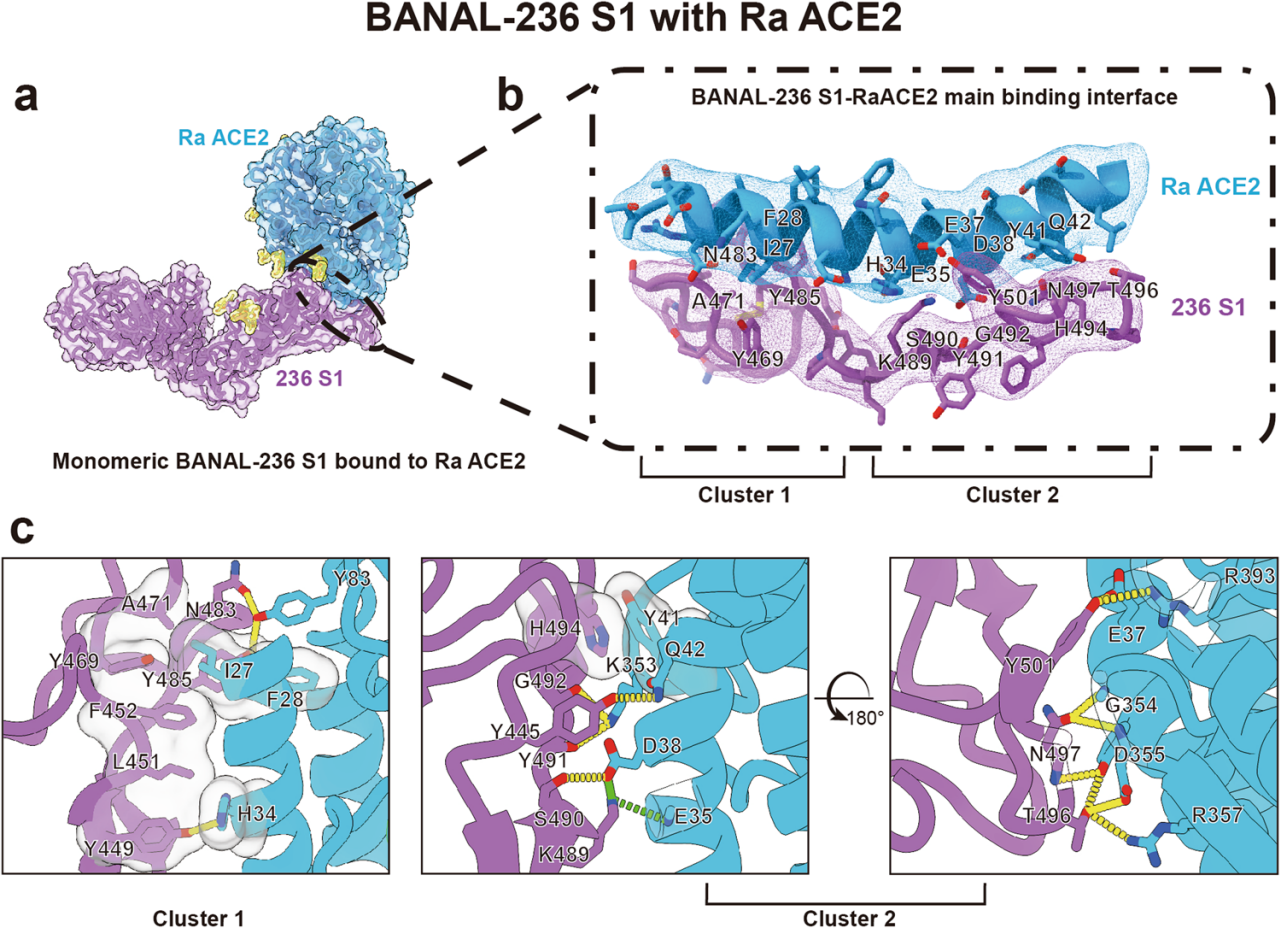
Cryo-EM structure of BANAL-20-236 S1 in complex with *R. affinis* ACE2. **a** Overall structure of BANAL-20-236 S1 in complex with *R. affinis* ACE2 (PDB ID: 8HXK). Purple: the BANAL-20-236 S1 in ribbon form; blue: *R. affinis* ACE2 in ribbon form. The surface presentation was semi-transparent. **b** Ribbon representation of the interaction interface of the BANAL-20-236 S1/Ra ACE2 complex. Purple: the BANAL-20-236 S1; blue: *R. affinis* ACE2. The EM map densities of BANAL-20-236 S1 and *R. affinis* ACE2 in B are shown in purple and blue meshes. There are two clusters of interaction at the interface, Clusters 1 and 2. **c** Detailed interaction between BANAL-20-236 S1 and *R. affinis* ACE2. Purple: the BANAL-20-236 S1; blue: *R. affinis* ACE2. Hydrogen bonds and salt bridges are presented as yellow dashed lines and green dashed lines, respectively, and hydrophobic interactions are shown as semi-transparent gray bubble-shape.

To gain further insights into the nature of interactions between BANAL S proteins and bat ACE2s, we selected ACE2 of *R. affinis* (RaACE2) for the structural study of S/ACE2 complex using cryo-EM, because RaACE2 showed the highest transduction efficiency among all bat ACE2s tested by both BANAL-20-52 and BANAL-20-236 S pseudovirions (Fig. 3a). The soluble RaACE2 proteins were mixed with the prefusion trimeric BANAL-20-236 ectodomain on ice for 15 min, and the pH was quickly changed to 5.5 before the mixture was snap-frozen for data collection by cryo-EM. The structure of only monomeric S1/RaACE2 complex was resolved at a resolution of 3.87 Å (Fig. 6a). The overall structure of BANAL-20-236 S1/RaACE2 resembles those of SARS-CoV-2 S/hACE2 and BANAL-20-236 RBD/hACE2 complexes. There are two interaction clusters at the interface of BANAL-20-236 S1/RaACE2 (Fig. 6a). While cluster 1 is dominated by hydrophobic interactions and formed by the interactions between I27, F28, H34, Y83 of RaACE2 and Y449, L451, F452, Y469, A471, N483, Y485 of BANAL-20-236 S proteins, the cluster 2 is mainly formed by hydrogen bonds and salt bridges and composed of E35, E37, D38, Y41, Q42, K353, G354, D355, R357, R393 of RaACE2 and Y445, K489, S490, Y491, G492, H494, T496, N497, Y501 of BANAL-20-236 S protein (Fig. 6b; Supplementary Fig. S16). Overall hydrophobic and hydrophilic interactions at the interface are similar between BANAL-20-236 S1/RaACE2 and BANAL-20-236 RBD/hACE2, but the contribution from individual residue varies significantly. For example, compared to hACE2, I27 of RaACE2 forms hydrophobic interactions with multiple residues of BANAL-20-236 S, including F452, Y469, A471, and Y485 (Fig. 6c); K489 in BANAL-20-236 S protein forms only one salt bridge with hACE2 but three hydrogen bond/salt bridges with RaACE2, and S490, which make no hydrophilic interaction with hACE2, forms three hydrogen bond/salt bridges with RaACE2 (Fig. 6c; Supplementary Fig. S16). Fewer residues are involved in hydrophilic interaction in BANAL-20-236 S1/RaACE2, compared to BANAL-20-236 RBD/hACE2, but the total numbers of hydrogen bond/salt bridges are similar, 27 for BANAL-20-236 S1/RaACE2 and 26 for BANAL-20-236 RBD/hACE2.

### Characterization of susceptibility of BANAL-20-52 and BANAL-20-236 in animals

**Fig. 7:**
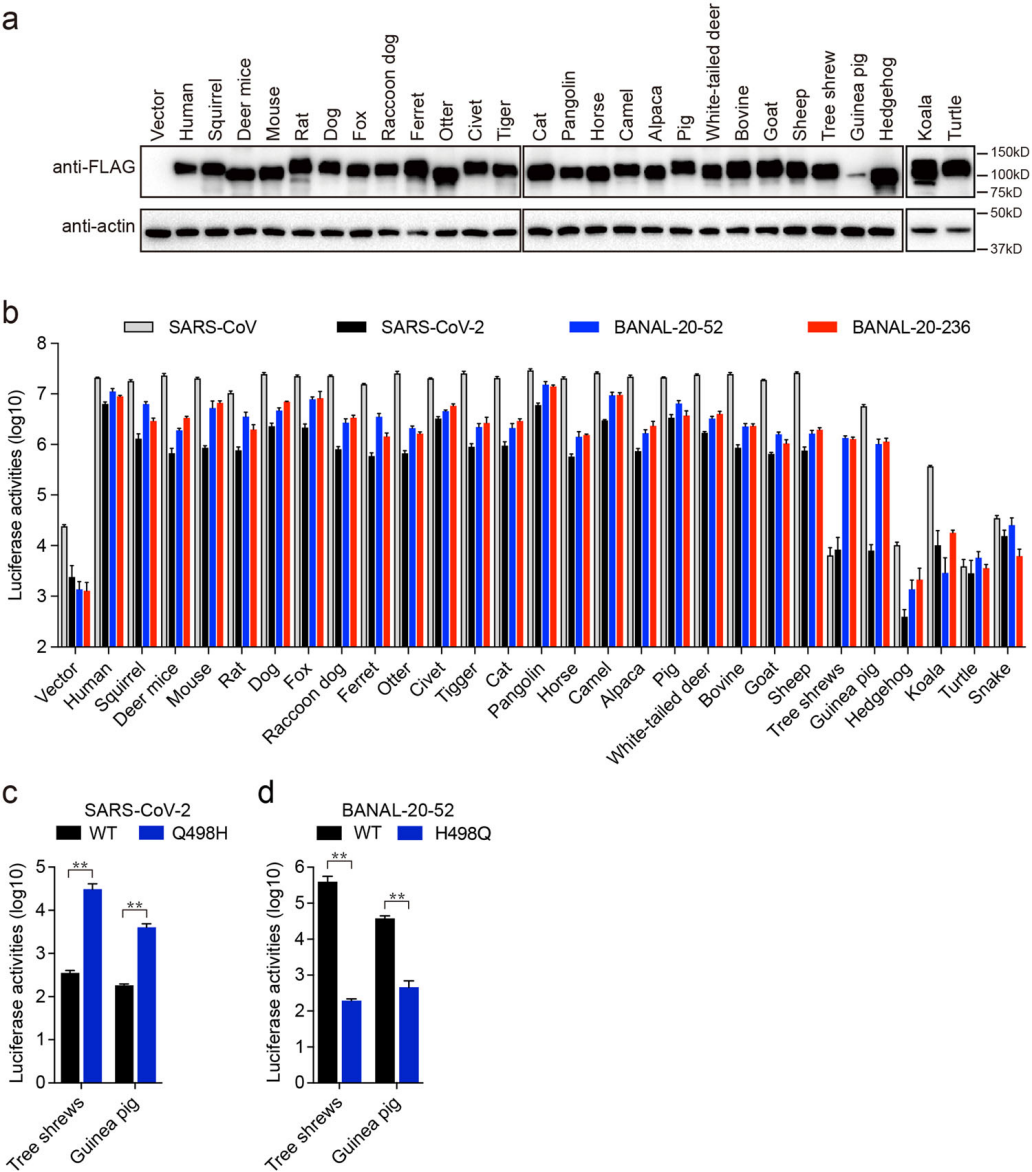
BANAL-20-52 and BANAL-20-236 S pseudovirions utilize a broad range of animal ACE2 orthologs for virus entry. **a** Western blot analysis of the cell lysates of HEK293 cells transiently over-expressing FLAG-tagged animal ACE2 orthologs using mouse monoclonal anti-FLAG M2 antibodies. β-actin served as loading controls. **b** Transduction of HEK293 cells expressing different animal ACE2 orthologs by SARS-CoV S, SARS-CoV-2 S, BANAL-20-52 S, and BANAL-20-236 S pseudovirions. The experiments were done in triplicate and repeated at least three times. One representative is shown with error bars indicating SD. **c**, **d** Transduction of 293 cells expressing tree shrew ACE2 or guinea pig ACE2 by WT and Q498H mutant SARS-CoV-2 S pseudovirions (**c**), and by WT and H498Q mutant BANAL-20-52 S pseudovirions (**d**). Data are represented as mean ± SD from at least triplicates. *P*-values in **c** and **d** are calculated by unpaired two-sided Student’s *t*-test. *P < 0.05; **P < 0.01; ns, *P* > 0.05.

Zoonotic transmission from bat to mammals to human has been proposed as one of the likely scenarios of the origination of SARS-CoV-2^35,36^, and recently we and others also showed that ACE2s from many mammal species were susceptible to bat SC2r-CoV RaTG13 transduction^14,15^. Next, we determined whether BANAL-20-52 and BANAL-20-236 S pseudovirions could use animal ACE2s for entry. Among ACE2s from twenty-six different animals, there are two from common pets (cat and dog), ten from domestic animals (ferret, horse, camel, alpaca, pig, bovine, goat, sheep, mouse, and guinea pig), fourteen from wild animals (squirrel, deer mice, rat, fox, raccoon dog, civet, otter, tiger, pangolin, white-tail deer, tree shrews, hedgehog, koala, and turtle), and turtle served as a non-mammal control. All animal ACE2s except for guinea pigs were expressed well (Fig. 7a) and transported to the cell surface (Supplementary Fig. S17) at levels similar to or higher than hACE2. Consistent with our previous report^14^, SARS-CoV and SARS-CoV-2 S pseudovirions showed very broad susceptibility among various mammal ACE2s tested, 23 out of 26 for SARS-CoV and 22 out of 26 for SARS-CoV-2 (Fig. 7b). Similar to SARS-CoV and SARS-CoV-2, both BANAL-20-52 and BANAL-20-236 also showed a potential broad animal host range. Except for hedgehog, koala, and turtle ACE2s, 23 animal ACE2s showed more than a 700-fold increase in luciferase activities than vector control by BANAL-20-52 and BANAL-20-236 S pseudovirions (Fig. 7b). Of note, tree shrews ACE2 was only susceptible to transduction by BANAL-20-52 and BANAL-20-236 S pseudovirions, and guinea pig ACE2 mediated highly efficient entry by SARS-CoV, BANAL-20-52 and BANAL-20-236 but not SARS-CoV-2 S pseudovirions (Fig. 7b), despite relative low expression compared to the other ACE2s. Since residue 498 of S protein plays a critical role in interaction with different bat ACE2s (Fig. 3e, f), we then asked whether this residue also affected the entry of S protein pseudovirions on tree shrews and guinea pig ACE2s or not. While Q498H mutation in SARS-CoV-2 increased virus entry on cells expressing tree shrews and guinea pig ACE2s by 87-fold and 22-fold, respectively (Fig. 7c), H498Q substitution almost abrogated entry on cells expressing tree shrews and guinea pig ACE2s by BANAL-20-52 S pseudovirions (Fig. 7d), indicating that residue 498 might also play a critical role in interacting with tree shrews and guinea pig ACE2s.

### Cross-immunogenicity among SARS-CoV-2, BANAL-20-52, and BANAL-20-236 S proteins

**Fig. 8:**
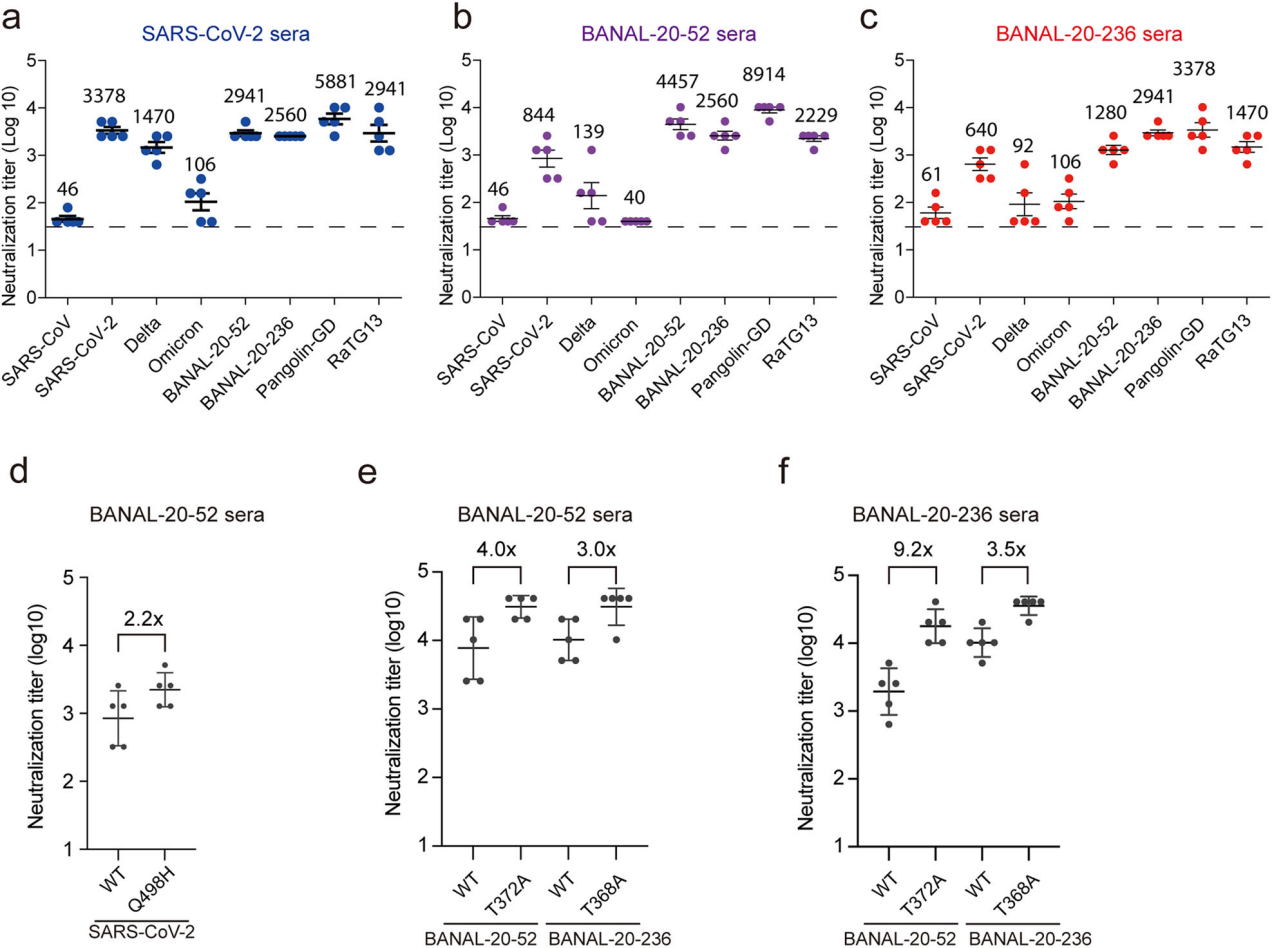
Cross-neutralization activities of sera from SARS-CoV-2, BANAL-20-52, and BANAL-20-236 S-immunized mice. **a**–**c** The NT_50_ against the indicated S pseudovirions of sera from mice immunized by trimeric SARS-CoV-2 S ectodomain (**a**), BANAL-20-52 S ectodomain (**b**), and BANAL-20-236 S ectodomain (**c**). The numbers above each group indicate the neutralizing GMTs of each group. **d** The NT_50_ against WT and Q498H mutant SARS-CoV-2 S pseudovirions of sera from trimeric BANAL-20-52 S ectodomain-immunized mice. **e**, **f** The NT_50_ against the indicated S pseudovirions of sera from mice immunized with trimeric BANAL-20-52 S ectodomain (**e**) or BANAL-20-236 S ectodomain (**f**).

The S protein is not only responsible for virus entry but also the primary target for vaccine development. Since both BANAL-20-52 and BANAL-20-236 have zoonotic transmission potential, we asked whether there were any cross-immunogenic activities among SARS-CoV-2 (WH strain), BANAL-20-52, and BANAL-20-236 S proteins. Mice were immunized with the trimeric S ectodomain proteins twice, with a 14 day interval between two immunizations. The sera were collected at day 28 post first immunization, and antibodies present in the sera were tested for their ability to bind the S proteins by ELISA using the homotypic S proteins as the bait and their ability to neutralize various sarbecoviruses was also evaluated using pseudotyped lentiviruses. All three S proteins were very immunogenic and induced high levels of S protein-binding antibodies against themselves (Supplementary Fig. S18). Sera from SARS-CoV-2 S-immunized mice showed high neutralization activities against itself (WH strain), BANAL-20-52, BANAL-20-236, pangolin-GD, and RaTG13 with the neutralizing geometric mean titers (GMT) at 3378, 2941, 5881, and 2941, respectively (Fig. 8a). Compared to the homotypic SARS-CoV-2 WH strain, the neutralizing GMTs against delta and omicron BA.1 variant were decreased by 2.3- and 31.9-fold, respectively, consistent with previous reports^37,38^. Sera from BANAL-20-52 S-immunized mice also showed very good neutralizing activity against itself, BANAL-20-236, pangolin-GD, and RaTG13 with GMTs at 4457, 2560, 8914, and 2229, respectively (Fig. 8b). In contrast, their neutralizing activities against various SARS-CoV-2 variants, including WH strain, delta, and omicron, were substantially reduced by more than 5-fold, 32-fold, and 100-fold, respectively, when compared to BANAL-20-52. Sera from BANAL-20-236-immunized mice showed neutralizing GMTs against itself and pangolin-GD at 2941 and 3378, respectively, whereas the neutralizing GMTs against BANAL-20-52 and RaTG13 were decreased by more than 2-fold (Fig. 8c). The GMTs of BANAL-20-236-immunized sera for all three SARS-CoV-2 variants tested, WH strain, delta, and omicron, were 640, 92, and 106, respectively, equivalent to 1/4.6, 1/32, and 1/27.7 of that of BANAL-20-236. BANAL-20-236 sera showed slightly better neutralizing GMT against omicron than delta, although both GMTs were relatively low. Of note, all immunized sera showed the highest neutralizing GMTs against pangolin-GD S pseudovirions, whereas they only showed minimal neutralizing activities against SARS-CoV S pseudovirions.

Sera from BANAL-20-52 S protein-immunized mice showed about a 5-fold difference in neutralizing GMTs between the SARS-CoV-2 WH strain and BANAL-20-52 (Fig. 8b). Since RBM is the main target of neutralization antibodies and residue 498 is the only difference in RBM between SARS-CoV-2 and BANAL-20-52, we reasoned that this might contribute to the difference in GMTs. The neutralizing GMT of BANAL-20-52 S-immunized mice sera against SARS-CoV-2 Q498H mutant virus was was 2560, about half of BANAL-20-52 and about 2.2-fold higher than SARS-CoV-2 (Fig. 8d), indicating that Q498H-specific antibodies might account for about 1/4 of neutralizing GMTs in BANAL-20-52-immunized mice sera. Since glycosylation in S protein may also affect the binding of neutralizing antibodies^39^ and T372A or T368A change in S protein likely removes one glycosylation, we then asked whether T372A or T368A change has any effect on the neutralizing GMT. Compared to WT, T372A substitution in BANAL-20-52 S increased the neutralizing GMTs of sera from BANAL-20-52 and BANAL-20-236 S-immunized mice by 4-fold and 9.2-fold, respectively (Fig. 8e, f), and T368A change in BANAL-20-236 also increased the neutralizing GMTs of BANAL-20-52 and BANAL-20-236 S-immunized mouse sera by 3-fold and 3.5-fold (Fig. 8e, f), respectively, indicating the potential selective advantage in immune evasion conferred by T372 over A372.

Since SARS-CoV-2 S-immunized mice sera showed good neutralizing GMTs against both BANAL-20-52 and BANAL-20-236 pseudovirus transduction, we then determined whether convalescent sera from recovered COVID-19 patients and sera from person vaccinated with inactivated SARS-CoV-2 vaccine could neutralize BANAL-20-52 and BANAL-20-236 pseudoviruses. Like SARS-CoV-2 S-immunized mouse sera, all human sera tested showed evident neutralizing activities against BANAL-20-52 and BANAL-20-236 pseudoviruses at levels similar to SARS-CoV-2 WH strain (Supplementary Fig. S19), indicating that current SARS-CoV-2 vaccine might be able to provide adequate protection against BANAL-20-52 and BANAL-20-236 infection.

## Discussion

There are more than 1400 bat species worldwide^40^, accounting for about 20% of all mammal species. Bats harbor many viral human pathogens, such as paramyxoviruses^41^, rabies virus^42^, lyssaviruses^43^, etc. Various CoVs have also been found in bats^10,44^ and several human CoVs have been thought to originate from bats, including SARS-CoV-2^5,45,46^, although the immediate ancestor of SARS-CoV-2 remains unknown. Bat CoVs BANAL-20-52 and BANAL-20-236 share over 95% sequence identity with SARS-CoV-2, and both can also use hACE2 for virus entry (Fig. 1)^13^, indicating that they might possess the risk of zoonotic transmission from bat to human directly or through an intermediate host. Therefore, it would be important to know the potential host ranges of these CoVs to prevent or minimize the risk of their emergence in humans. Indeed, both BANAL-20-52 and BANAL-20-236 showed a very broad host range of susceptibility among various bats and animal species (Figs. 3 and 7). All 14 bat ACE2s and 23 out of 26 animal ACE2s we tested are susceptible to infection mediated by both S proteins, indicating that both bat CoVs might have extensive host ranges and pose a high risk of potential zoonotic transmission.

BANAL-20-52 was first detected in *R. malayanus* bat and could use ACE2 of *R. malayanus* bat for virus entry (Fig. 3b). However, its S protein transduction efficiency on *R. malayanus* bat ACE2 was quite limited, even about 10-fold lower than BANAL-20-236 (Fig. 3b). Although we could not rule out the possibility that the low transduction on this *R. malayanus* ACE2 by BANAL-20-52 pseudovirions might result from specific ACE2 polymorphism among different *R. malayanus* bats, the low infection raises the question of whether there might be other bat species as the possible natural host for BANAL-20-52 virus or not, and this notion is further supported by the higher susceptibility among other bat species by BANAL-20-52 (Fig. 3b). BANAL-20-236 was originally found in *R. marshalli* bat. Unfortunately, till now, we could not find any sequence information of *R. marshalli* ACE2 available in the Genbank. However, the broad susceptibility of BANAL-20-236 among different bat ACE2s indicates that the natural host ranges for BANAL-20-236 may likely be beyond *R. marshalli* bats. Indeed, host-switching is frequently found among some bat CoVs^47^ and different bat species contain some bat CoVs with nearly 100% identical viral genomes^10^. Of note, all thirteen bat species in this study are known to harbor sarbecoviruses^5–10,13,26–28^, and nine out of thirteen (except for *H. pratti*, *P. abramus*, *R. ferrumequinum*, and *R. sinicus*) are commonly habituating in Indochina peninsula and southern China (Supplementary Fig. S20)^10,48^. Of particular interest, ACE2s of *R. affinis*, *R. luctus*, *R. malayanus*, and *R. pussilus* can efficiently mediate entry of BANAL-20-52 and BANAL-20-236, and their habitats and roosting behaviors also share high similarities^10,48^, which might result in host-switching of some bat CoVs. Further surveillance of bat CoVs among various bat species is urgently warranted.

Depending on CoV and bat species, the transmission route of CoVs in bats may vary, but the fecal-oral route always plays a very important role in CoV transmission^31,32^. One main obstacle to the fecal-oral transmission of CoVs is to survive through relatively rough conditions in the bat stomachs, including protease digestion and relatively low pH at about 5.6^33^ before reaching the intestine where CoVs infect and propagate. We hypothesized that the selective pressure exerted by the fecal-oral transmission route might prefer the S protein in all “down” conformation to increase its stability for surviving the passage through the stomach. Indeed, we found that, like RaTG13 S proteins^49,50^, S proteins of BANAL-20-52 and BANAL-20-236 in pH 5.5 are also folded in a relatively compact-all "down" conformation (Fig. 2), which might not only increase the S protein stability but also minimize the exposure of potential protease cleavage sites. The augmented S protein stability is supported by over 6000 Å^2^ of the overall interface interaction areas between monomers of S proteins of BANAL-20-52 and BANAL-20-236, about 1300 Å^2^ more than those of respiratory-transmitted omicron BA.2.75 (Fig. 5c), by the well-structured 630 and FPPR loops (Fig. 5d), and by bolt-like extra NAG modification at residue 370 (Fig. 5e), together keeping S protein in all “down” conformation. Moreover, despite that T372A change markedly increases viral infectivity over WT^30^ (Fig. 4a), all known SC2r-CoVs retain a T372 or S372 in the S protein. Limited trypsin digestion at pH 5.5, a condition roughly mimicking bat stomach condition, reveals that T372A substitution might render some RBDs to assume the “up” conformation, facilitating the dissociation of S1 subunits from S proteins and resulting in loss of infectivity after trypsin treatment. In contrast, the cleaved WT S1 subunits are stably associated with the S protein (Fig. 4h, i; Supplementary Fig. S12), and viruses remain infectious (Fig. 4). The relative resistance to protease digestion likely contributes to the selective advantage of T372 over A372 in S proteins during natural selection, in agreement with our hypothesis that virus stability might be more favored in transmission through the fecal-oral route than infectivity. Finally, we also found that the A372 pseudovirus is more sensitive to homotypic and heterotypic neutralization than the T372 virus (Fig. 8e, f), suggesting that the selective advantage in immune evasion might also play in favor of T372 over A372 during evolution.

Multiple residues, including 453, 493, 498, and 501, etc.^14,51–53^ in the RBD of SARS-CoV-2 S protein, plays critical roles in interaction of multiple animal ACE2s. Residue 498, especially H498, has been implicated in recognition of pangolin, mouse, rat, and European hedgehog ACE2s^14,54^. Indeed, in this study, we further found that H498 might also be crucial for interaction with tree shrews, guinea pig, *R. malayanus, R. sinicus* HB, and *P. abramus* ACE2s (Fig. 3, Fig. 7), indicating that H498 might be advantageous for extensive host ranges and close attention should be paid to bat CoVs harboring H498 in their S protein. H498 (or H494 in BANAL-20-236) forms strong hydrophobic interaction with a highly conserved Y41 in ACE2 (Supplementary Fig. S10), which might enhance S/ACE2 interaction leading to the expansion of the host range. H498 is also present in most of pangolin SC2r-CoVs. However, as of April 6th, 2023, there are only 111 genomes with Q498H mutation out of over 13.92 million SARS-CoV-2 complete genomes collected in the GISAID database, indicating that H498 SARS-CoV-2 virus might not have a fitness advantage in humans. In contrast, most of the omicron variants, which currently dominate global circulation of SARS-CoV-2, contain a Q498R mutation and show extended host susceptibility on mice and Pearson’s horseshoe bats^55^. However, N501Y, another mutation associated with a broad host range, is also present in the omicron genomes, and Q498R/N501Y double mutation might increase the binding affinity between S and ACE2^56^. Whether Q498R alone contributes to the extended host range warrants further investigation. The residue 498 also appears to be one of the important immunogenic sites, and the exact amino acid in this position might influence its immunogenicity. Q498 might be less immunogenic since SARS-CoV-2 S sera showed similar GMTs against SARS-CoV-2 and BANAL-20-52 pseudoviruses, whereas H498 seems to be highly immunogenic since antibodies against H498 might be responsible for about 25% of total neutralizing activities against SARS-CoV-2 H498 pseudoviruses in BANAL-20-52 S-immunized mice sera (Fig. 8d).

The presence of furin site between S1 and S2 subunits of S protein has been attributed to the enhancement of virus entry on primary human airway epithelial cells and CaLu3 cells and the increase of transmissibility of SARS-CoV-2 in humans and animals^57^. Cleavage at S1/S2 conjunction by furin facilitates the activation cleavage at S2’ site by TMPRSS2 or other host proteases, resulting in the increase of membrane fusion on the cell surface and virus entry^58,59^. Indeed, adding an exogenous furin site in BANAL S proteins significantly increases transduction on CaLu3 cells. However, NTD seems also to play an important role in the entry of CaLu3 cells (Fig. 1). While NTD of BANAL-20-52 facilitates entry of CaLu3 cells, S proteins with NTD of BANAL-20-236 shows substantial reduction in virus infectivity on CaLu3 cells (Fig. 1). NTDs might affect either efficiency of TMPRSS2 cleavage and conformational changes of S protein^60^ or binding of different attachment factors, which might be present on 293/hACE2 and CaLu3 cells at different levels. However, more research is warranted to shed light on the exact mechanism.

This study determines the susceptibility of two SC2r-CoVs among various bat and animal species. However, virus entry is only the first step of the virus life cycle; post-entry block could restrict virus infection and make the host non-permissive. Moreover, the pH 5.6 of bat stomach is based on different insectivorous bat species; whether it might be true for other individual *rhinolophus* bat species remains to be determined.

In conclusion, we determined the potential host ranges of two bat SC2r-CoVs and solved the cryo-EM structures of their S proteins and the BANAL-20-236 S1/RaACE2 complex. More importantly, we found that the all “down” and compact structure of S proteins of bat CoVs confer selective advantage in the fecal-oral transmission during virus evolution.

## Materials and methods

### Animals

Female 6- to 8-week-old BALB/c mice were ordered from Beijing Vital River Laboratory Animal Technology Co., Ltd. All the procedures related to animal handling, care, and treatment were approved by the Animal Use and Care Committee of the Institute of Laboratory Animal Science, Chinese Academy of Medical Sciences & Peking Union Medical College (GH20002).

### Human sera

Human serum samples were collected from 2 COVID-19 convalescent patients and 4 vaccinated individuals who received two doses of inactivated vaccines (CoronaVac Sinovac Biotech or BBIBP-CorV Sinopharm). All volunteers signed informed consent forms. The protocol of this study was approved by the Ethic Review Board of Institute of Pathogen Biology, Chinese Academy of Medical Sciences & Peking Union Medical College (IPB-2020-01).

### Cell lines

HEK293T cells (ATCC CRL-3216), HEK293 cells (ATCC CRL-1573), HEK293 cells stably expressing hACE2 (293/hACE2 cells) and human airway epithelial CaLu3 cells (ATCC HTB-55) were cultured in Dulbecco’s modified Eagle’s medium (DMEM) supplemented with 10% fetal bovine serum (FBS) and 1% penicillin-streptomycin-fungizone (PSF) at 37 °C with 5% CO_2_. Suspension Expi293F cells were cultured in Expi293 expression medium (Gibco A14527) and incubated at 37 °C with 5% CO_2_ with orbital shaking at 125 rpm.

### Plasmid constructions and site-directed mutagenesis

The codon-optimized BANAL-20-52 S gene (GISAID EPI_ISL_4302644), BANAL-20-236 S gene (GISAID EPI_ISL_4302647), Pangolin-GD S gene (GenBank MT799521.1), RaTG15 S gene (NGDC GWHBAUP01000000), SARS-CoV-2 Delta variant S gene (GISAID EPI_ISL_3940074) and Omicron variant S gene (GISAID EPI_ISL_6640919) with a deletion of the last 19 amino acids were synthesized by GenScript (Nanjing, China) and cloned into p3×FLAG-CMV-14 vector between *Hind*III and *Xba*I sites to generate p3×FLAG-CMV14-BANAL-20-52-S-delta19, p3×FLAG-CMV14-BANAL-20-236-S-delta19, p3×FLAG-CMV14-Pangolin-GD-S-delta19, p3×FLAG-CMV14-RaTG15-S-delta19, p3×FLAG-CMV14-Delta-S-delta19 and p3×FLAG-CMV14-Omicron-S-delta19, respectively. Plasmids encoding SARS-CoV, SARS-CoV-2 and RaTG13 S protein with a deletion of the last 19 amino acids were described previously^14,16^. The DNA fragments encoding BANAL-20-52 S ectodomain (residues 1–1204, K982P and V983P mutants) and BANAL-20-236 S ectodomain (residues 1–1200, K978P and V979P mutants) with a C-terminal bacteriophage T4 fibritin foldon trimerization motif followed by an 8× His tag and a twin-strep tag were subcloned into pcDNA3.1(+) between *Hind* III and *Xba* I sites to generate pcDNA3.1-BANAL-20-52-S-trimer and pcDNA3.1-BANAL-20-236-S-trimer plasmids, respectively. The DNA fragments for SARS-CoV-2 RBD (residues 319-541), BANAL-20-52 RBD (residues 319-541), BANAL-20-236 RBD (residues 315–537), and soluble *R.affinis* ACE2 (residues 19–615) with an N-terminal signal peptide and a C-terminal twin-strep tag were subcloned into pcDNA3.1(+) to generate pcDNA3.1-SARS-CoV-2-RBD, pcDNA3.1-BANAL-20-52-RBD, pcDNA3.1-BANAL-20-236-RBD, and pcDNA3.1-sRaACE2 plasmids, respectively. All bat and animal ACE2 coding genes were synthesized by GenScript (Nanjing, China) according to reference sequences and cloned into p3×FLAG-CMV-14 between *Hind*III and *Xba*I sites. The lentiviral packaging plasmid psPAX2 was obtained from Addgene (Cambridge, USA). The pLenti-GFP lentiviral reporter plasmid that expresses GFP and luciferase was generously gifted by Dr. Fang Li (Duke University). All mutants were produced by site-directed mutagenesis. After the entire coding sequences were verified by sequencing, the fragments were subcloned back into the corresponding vectors.

### Production and transduction of S-pseudotyped lentiviruses

Lentiviruses pseudotyped with different coronavirus S proteins were produced as described previously with minor modifications^16^. Briefly, plasmids encoding different coronavirus S proteins were co-transfected with pLenti-GFP and psPAX2 into HEK293T cells at a molar ratio of 1:1:1 by using PEI. After 40 h of incubation, the supernatant media containing pseudovirions were centrifuged at 1000× *g* for 10 min to remove cell debris. To quantify the transduction efficiency of S-pseudotyped lentiviruses, the target cells were seeded in a 24-well plate at about 30%–40% confluence. After 24 h incubation, the cells were inoculated with S-pseudotyped lentiviruses. For inhibitor assays, cells were pre-treated with drugs at 37 °C for 1 h and then transduced with pseudovirions in the presence of different inhibitors. At 40 h post-inoculation, the cells were lysed using Steady-Glo Reagent (Promega, Madison, WI). Transduction efficiency was monitored by quantitation of luciferase activity using Modulus II Microplate Reader (Turner BioSystems, Sunnyvale, CA, USA). All experiments were done in triplicate and repeated at least three times.

### Detection of viral spike glycoproteins by western blot assay

To evaluate S protein expression in cell lysates, HEK293T cells were transfected with plasmids encoding different coronavirus S proteins using PEI. Forty hours post-transfection, the cells were lysed using RIPA lysis buffer (50 mM Tris-HCl, pH 7.4, 150 mM NaCl, 1% NP-40, 0.1% SDS, 1 mM EDTA, and 1% sodium deoxycholate) with protease inhibitors (Selleck). To analyze the level of S proteins in pseudovirions, the pseudovirus-containing supernatant was pelleted through a 20% sucrose cushion at 25,000 rpm at 4 °C for 2 h in a Beckman SW41 rotor. Viral pellets were resuspended in 1× loading buffer. Cell lysates and pseudovirion pellets were then separated on a 10% SDS-PAGE and transferred to a nitrocellulose blot. The S proteins were detected with rabbit polyclonal anti-S2 antibody (1:2000) or mouse monoclonal anti-FLAG M2 antibody (1:2000). The β-actin and HIV capsid protein (p24) were detected using mouse monoclonal anti-β-actin antibody (1:5000) (Sigma, St Louis, MO, USA) and rabbit polyclonal anti-p24 antibody (1:5000) (Sinobiological Inc, Beijing, China), respectively. The blots were further stained with horseradish peroxidase-conjugated goat anti-rabbit IgG or goat anti-mouse IgG, and then visualized with Clarity Western ECL substrate (Bio-Rad, Hercules, CA, USA).

### Cell–cell fusion assay

HEK293T cells were co-transfected with the plasmids encoding different CoV S proteins and the plasmids encoding GFP. After 40 h incubation, the cells were detached with 0.25% trypsin for 2 min and overlaid on a 70% confluent monolayer of 293/hACE2 cells at a ratio of 1:2. After 4 h of incubation, images of syncytia were captured with a Nikon TE2000 epifluorescence microscope running a MetaMorph software (Molecular Devices).

### Mouse immunization and serum collection

Female 6- to 8-week-old BALB/c mice were randomly divided into four groups (control, SARS-CoV-2 S-trimer, BANAL-20-52 S-trimer, and BANAL-20-236 S-trimer). For vaccination, mice were immunized intraperitoneally with 5 μg of trimeric SARS-CoV-2 S ectodomain, BANAL-20-52 S ectodomain, or BANAL-20-236 S ectodomain proteins with aluminum hydroxide adjuvant (0.5 mg/mL aluminium hydroxide, 4 mM phosphate, 0.85% NaCl) on day 0 and day 14. The mice mock immunized with PBS were used as controls. Sera were collected on day 28 post-immunization. All sera were heat-inactivated at 56 °C for 30 min after collection and stored at –80 °C for reuse.

### Measurement of serum IgG binding to RBD by ELISA

All immunized mouse serum samples were heat-inactivated at 56 °C for 30 min before use. Briefly, 96-well plates (Beijing Wantai Biological Pharmacy, Beijing, China) were coated overnight at 4 °C with 0.5 µg/well of purified SARS-CoV-2, BANAL-20-52, or BANAL-20-236 RBD proteins in PBS. After blocking with 5% fat-free dry milk in TBS-T (blocking buffer) for 1 h at room temperature, 5-fold serially diluted heat-inactivated sera (starting dilution 1:100) in blocking buffer were added to the plates. After 1 h incubation, the plates were washed three times, and horseradish peroxidase HRP-conjugated goat anti-mouse IgG in blocking buffer at a dilution of 1:5000 was added to the plates and incubated at room temperature for 1 h. After washing three times, 100 μL of 3,3′,5,5′-Tetramethylbenzidine (TMB) substrate (Beijing Wantai) was added into each well and incubated in the dark at room temperature for 15 min. The reaction was then stopped with 50 μL of stop solution (Beijing Wantai), and the absorbance at 450 was recorded using a MultiSkan MK3 plate reader (Thermo, Rochester, NY, USA). The IgG endpoint GMTs were defined as the dilution fold, which emitted an optical density exceeding 2× background (without serum but the secondary antibody was added).

### Pseudovirus-based neutralization assay

Lentivirues pseudotyped with different coronavirus S proteins were pre-incubated with 2-fold serially diluted immunized mouse sera (starting at 1:40) or human sera (starting at 1:20) for 1 h at 37 °C, and then virus–antibody mixture was added onto 293/hACE2 cells in 96-well plates. After overnight incubation, the inoculum was replaced with a fresh medium. Cells were lysed 40 h later and pseudovirus transduction was measured as previously described. The 50% neutralization titer (NT_50_) values were defined as the farthest dilution that achieved more than 50% inhibition of pseudovirus infection compared with the control group. All experiments were done in triplicate.

### Protein expression and purification

Expression and purification of the S trimers were carried out as previously described^14^. Briefly, Expi293F cells (Gibco) were transiently transfected with the plasmids encoding different trimeric S proteins using Expifectamine 293 Transfection kit (Life Technologies, USA) according to manufacturer’s instructions. Cell culture supernatants were collected 3 d later and purified using Strep-Tactin XT Superflow high-capacity resin (IBA Lifesciences). After washing with 10-bed volumes of wash buffer (20 mM Tris, 200 mM NaCl, pH 8.0), the S trimers were eluted by the elution buffer (wash buffer with 50 mM biotin) (Sigma). After elution, the elution buffer was replaced with sodium citrate buffer (0.1 M sodium citrate, pH 5.5) using a centrifugal filter (Millipore), immediately followed by negative staining analysis. For expression and purification of soluble ACE2s or RBD proteins, the procedures were similar to above except that the elution buffer was replaced with the wash buffer (20 mM Tris, 200 mM NaCl, pH 8.0).

### Cell surface protein biotinylation assay

The cell surface biotinylation procedure has been described previously^14^. Briefly, HEK293 cells transiently expressing FLAG-tagged ACE2s were incubated with PBS containing 2.5 μg/mL EZ-linked Sulfo-NHS-LC-LC-biotin (Thermo-Pierce, Waltham, MA, US) on ice for 30 min after washing with ice-cold PBS. The reaction was then quenched using PBS with 100 mM lysine, and the cells were lysed with RIPA buffer. To pull down the biotin-labeled proteins, the lysates were incubated with NeutrAvidin beads (Thermo-Pierce, Waltham, MA, US) overnight at 4 °C. After washing 3 times with RIPA buffer, samples were resuspended in 30 μL of loading buffer and boiled for 10 min, and the level of ACE2 expression was determined by western blot assay using an anti-FLAG M2 antibody (1:2000). The β-actin and integrin β1 were used as loading controls for input and biotinylation, respectively.

### Proteolytic stability of S-pseudotyped lentiviruses

Pseudovirus-containing supernatant was pelleted through a 20% sucrose cushion at 25,000 rpm at 4 °C for 2 h in a Beckman SW41 rotor. Viral pellets were resuspended in DMEM with low pH (pH 5.5). The pseudovirions were then treated with serial trypsin concentrations for 10 min or 2.5 μg/mL trypsin for different time periods at pH 5.5. The reaction was then stopped with soybean trypsin inhibitors. The protease-treated pseudovirions were then used for transduction and western blot analysis. For western blot analysis, the pesudovirions were first centrifugated at 15,000 rpm for 2 h. After centrifugation, supernatants and pellets were separated. The S1 subunits in supernatants and pellets were then detected by western blot analysis using rabbit polyclonal anti-SARS-CoV-2 RBD antibodies.

### Cryo-EM sample preparation and data collection

BANAL-20-236/52 S-trimer: R1.2/1.3 300-mesh Quantifoil grids were glow-discharged for 2 min at 50 W. Then 3.5 μL of the BANAL-20-236/52 S-trimer protein (buffer: 100 mM sodium citrate, pH 5.5) at 1 mg/mL was applied on a pre-glow-discharged holey carbon-coated gold grid (Quantifoil, 300-mesh, R1.2/1.3), blotted for 7 s with no force in 100% humidity at 4 °C. After that the gold grid was immediately plunged into the liquid ethane by Vitrobot (FEI). Cryo-EM datasets of samples were collected on an FEI Titan Krios microscope operated at 300 kV. Movies (32 frames, each 0.2 s, accumulated dose of 60 e^−^Å^−2^) were recorded using a K2 detector with a defocus range between 1.4–2.2 μm by SerialEM yielding a final pixel size of 1.04 Å.

BANAL-20-236 S1 in complex with *R. affinis* ACE2: R1.2/1.3 300-mesh Quantifoil grids were glow-discharged for 2 min at 50 W. The BANAL-20-236 S-trimer protein and *R. affinis* ACE2 were mixed in a 1:4 ratio at pH 7.5 (buffer: 20 mM Tris, pH 7.5, 200 mM NaCl) and incubated on ice for 10 to 20 min. Then the buffer of the mixture sample was changed to pH 5.5 (buffer: 100 mM sodium citrate, pH 5.5). After that, 3.5 μL of the mixed sample was applied on a pre-glow-discharged holey carbon-coated gold grid (Quantifoil, 300-mesh, R1.2/1.3), blotted for 7 s with no force in 100% humidity at 4 °C. The gold grid was immediately plunged into the liquid ethane by Vitrobot (FEI). Cryo-EM datasets of samples were collected on an FEI Titan Krios microscope operated at 300 kV. Movies (32 frames, each 0.2 s, accumulated dose of 60 e^−^Å^−2^) were recorded using a K3 detector with a defocus range between 1.4–2.2 μm by SerialEM yielding a final pixel size of 1.07 Å.

### Cryo-EM data processing

All the micrographs were processed with MotionCor2^61^ in Relion3.0^62,63^, followed by contrast transfer function (CTF) estimation using Gctf^64^. Then particles of BANAL-20-236/52 S-trimer and BANAL-20-236 S1 in complex with *R. affinis* ACE2 were picked and extracted by Relion3.0 (Supplementary Fig. S6).

BANAL-20-236/52 S-trimer: reference-free 2D alignment by Relion3.0 was applied for BANAL-20-236/52 S-trimer particles. High-quality particles were selected and the species containing BANAL-20-236/52 all-closed trimeric spike proteins were separated by template-guided 3D-classification in Relion3.0. The results yielded only one configuration with all RBD down, apart from the rubbish class. For all the classifications, no symmetry was imposed. After 3D classification, particles with good qualities were selected for auto-refinement with C3 symmetry and postprocessing in Relion3.0 to generate the final cryo-EM maps. For BANAL-20-52 all-closed S trimer, particles were subjected to Bayesian polishing in Relion3.0^65^ before the final refinement to improve the resolution.

BANAL-20-236 S1 in complex with *R. affinis* ACE2: particles were subjected to multiple rounds of two-dimensional classification using cryoSPARC^66^. The 2D classes that displayed a clear secondary structure were retained and split into two distinct subsets, which either resembled all-closed S trimers or S1 monomer in complex with ACE2. The subsets of S1 monomer in complex with ACE2, like the telephone handset, then were separated by 3D-classification in Relion3.0. The results yielded two similar configurations with S1 monomer in complex with ACE2, apart from other rubbish class. After that, two classes of particles with good qualities were selected for auto-refinement with no symmetry imposed and postprocessing in Relion3.0 to generate the final cryo-EM maps. Also of note, particles were subjected to Bayesian polishing in Relion3.0 before the final refinement to improve the resolution.

### Model fitting and refinement

The atom models of BANAL-20-236/52 S-trimer and BANAL-20-236 S1 in complex with *R. affinis* ACE2 were generated by first fitting the chains of native apo SARS-CoV-2 S-trimer (PDB ID: 6VXX)^67^ and dissociated S1 domain of Alpha Variant SARS-CoV-2 Spike bound to ACE2 (PDB ID: 7R10)^68^ into the obtained cryo-EM densities by Chimera^69^. Then the structure was manually adjusted and corrected according to the protein sequences and cryo-EM densities in Coot^70^, and finally, real-space refinement was performed by Phenix^71^. Details of the refinement statistics of the complexes are summarized in Supplementary Tables S5–S8.

### Molecular dynamics stimulation

Models of the RBD from BANAL-20-236/52, WT SARS-CoV-2 and their variants in complex with *P. abramus* ACE2 were firstly referred to the cryo-EM structure of SARS-CoV-2 spike RBD bound with hACE2 (PDB ID: 6M0J)^72^ and then checked with the WHAT IF web interface (https://swift.cmbi.umcn.nl/) to remove atomic clashes. After that, the structures were simulated with GROMACS-2021^73^. In brief, the OPLS force field with TIP3P water model was selected to prepare the dynamic system. Na^+^ and Cl^−^ ions were added into the system to make the system electrically neutral. Then, energy minimization using the steepest descent algorithm was carried out until the maximum force of 1000 kJ/mol was achieved. NVT ensemb1e via the Nose-Hoover method at 300 K and NPT ensemble at 1 bar with the Parinello-Rahman algorithm were employed successively to make the temperature and the pressure equilibrated, respectively. Finally, molecular dynamics production runs of 10 ns were performed with random initial velocities and periodic boundary conditions. The non-bonded interactions were treated using Verlet cut-off scheme, while the long-range electrostatic interactions were treated using particle mesh Ewald method. The short-range electrostatic and van der Waals interactions were calculated with a cut-off of 12 Å. All six models were simulated in the same protocol.

### Quantification and statistical analysis

All data were presented as means ± SD and analyzed using GraphPad Prism software version 9.3.1 unless otherwise indicated in figure legends, and unpaired two-sided Student’s *t-*test was performed for comparison, *P < 0.05; **P < 0.01; ns, *P* > 0.05. In general, at least two independent biological replicates were carried out for each experiment.

### Supplementary information

Supplementary Information

## Data Availability

All data and materials presented in this manuscript are available from the corresponding author (Z.Q.) upon a reasonable request under a completed Materials Transfer Agreement (MTA). The cryo-EM maps have been deposited to Protein Data Bank (PDB) under the accession codes: 8HXJ (BANAL-20-52 S-trimer), 8I3W (BANAL-20-236 S-trimer), and PDB: 8HXK (BANAL-20-236 S1/RaACE2 complex) and are publicly available as of the date of publication. Any additional information required to re-analyze the data reported in this study is available from the lead contact upon request.

## Material availability

All materials and reagents generated in this study are available from the lead contact with a completed MTA.
