# Author Correction: Host susceptibility and structural and immunological insight of S proteins of two SARS-CoV-2 closely related bat coronaviruses

**DOI:** 10.1038/s41421-023-00597-1

**Published:** 2023-10-09

**Authors:** Xiuyuan Ou, Ge Xu, Pei Li, Yan Liu, Fuwen Zan, Pan Liu, Jiaxin Hu, Xing Lu, Siwen Dong, Yao Zhou, Zhixia Mu, Zhiqiang Wu, Jianwei Wang, Qi Jin, Pinghuang Liu, Jian Lu, Xiangxi Wang, Zhaohui Qian

**Affiliations:** 1 Key Laboratory of Pathogen Infection Prevention and Control (Peking Union Medical College), Ministry of Education, Beijing, China; 2 NHC Key Laboratory of Systems Biology of Pathogens, Institute of Pathogen Biology, Chinese Academy of Medical Sciences & Peking Union Medical College, Beijing, China; 3 State Key Laboratory of Respiratory Health and Multimorbidity, Chinese Academy of Medical Sciences & Peking Union Medical College, Beijing, China; 4 CAS Key Laboratory of Infection and Immunity, National Laboratory of Macromolecules, Institute of Biophysics, Chinese Academy of Sciences, Beijing, China; 5 Key Laboratory of Animal Epidemiology of the Ministry of Agriculture, College of Veterinary Medicine, China Agricultural University, Beijing, China; 6 College of Life Sciences, Peking University, Beijing, China

**Keywords:** Cryoelectron microscopy, Mechanisms of disease

Correction to: *Cell Discovery* (2023) 9:78

10.1038/s41421-023-00581-9 published online 28 July 2023

During the final submission of this manuscript, we inadvertently misplaced the Supplementary Fig. S4 as the Supplementary Fig. S5. The correct Supplementary Fig. S5 is shown as below. We are sorry for any inconveniences that might cause.
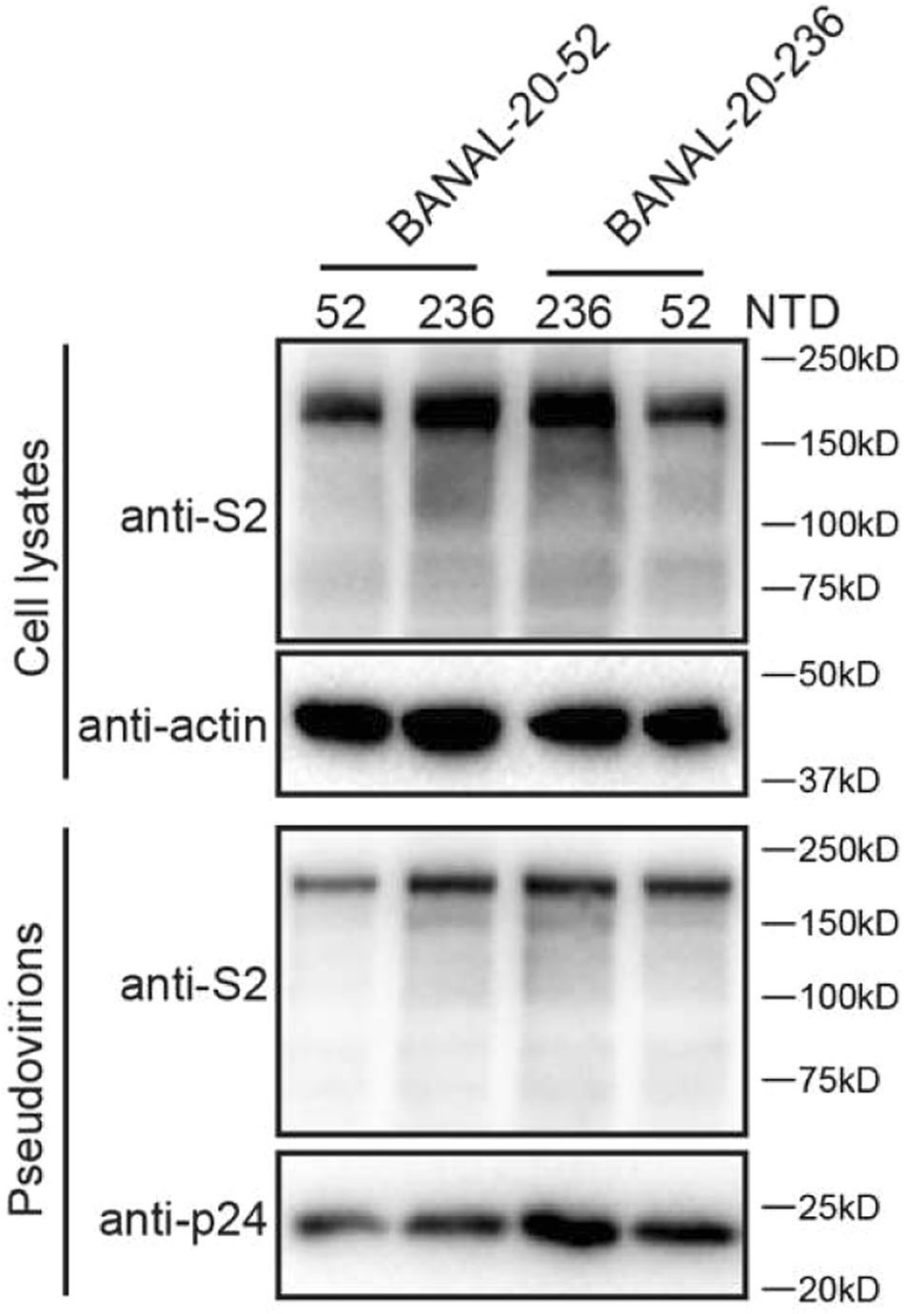


### Supplementary information

new supplementary file

