# Supplementary material for: Host susceptibility and structural and immunological insight of S proteins of two SARS-CoV-2 closely related bat coronaviruses
Source: Cell Discov. 2023 Jul 28;9:78. doi: 10.1038/s41421-023-00581-9 (PMC10382498; doi:10.1038/s41421-023-00581-9)
Supplement: Supplementary file 1 — Supplementary Information [file 41421_2023_581_MOESM1_ESM.pdf]

Supplementary information

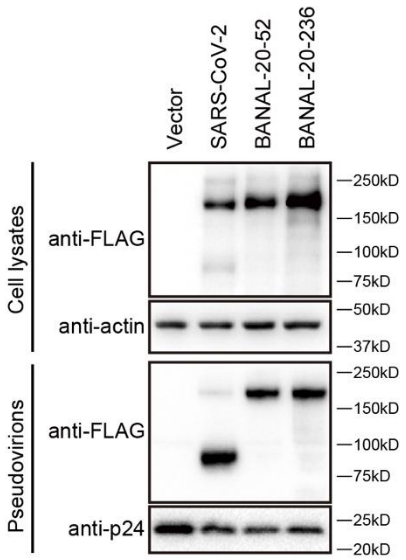

Supplementary Fig. S1 Western blotting of SARS-CoV-2 S, BANAL-20-52 S and BANAL-20-236 S proteins. The S protein was detected using mouse monoclonal anti-FLAG M2 antibody. β-actin and gag-p24 served as loading controls (cell lysates, top panel; pseudovirions, bottom panel).

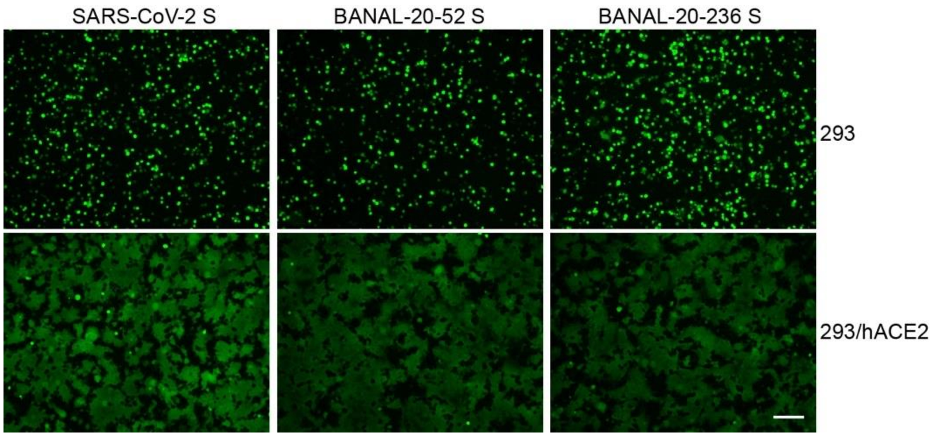

Supplementary Fig. S2 Cell-cell fusion mediated by the S proteins of SARS-CoV-2, BANAL-20-52, and BANAL-20-236. HEK293T cell transiently overexpressing S proteins and GFP protein were lifted with trypsin and then overlaid on 293/hACE2 cells. After 3 hours of co-culturation, images of cell-cell fusion were captured. Scale bar, 100 μM.

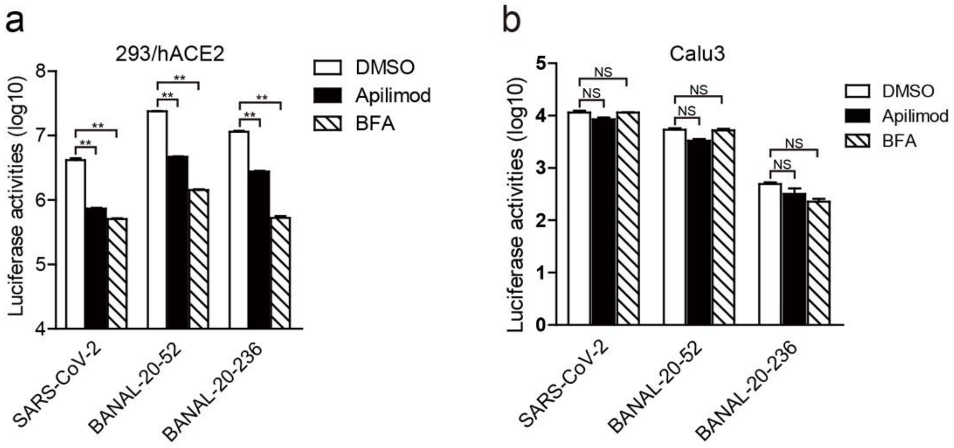

Supplementary Fig S3. Inhibition of entry of SARS-CoV-2 S, BANAL-20-52 S, and BANAL-20-236 S pseudovirions by apilimod and bafilomycin A1. (a and b) Inhibition of entry of SARS-CoV-2 S, BANAL-20-52 S, and BANAL-20-236 S pseudovirions into 293/hACE2 (a) and Calu3 (b) cells by the PIKfyve inhibitor apilimod (API) or the vacuolar H+-ATPase inhibitor bafilomycin A1(BFA).

Data are represented as mean ± standard deviation (SD) from at least triplicates.

p values in (a) and (b) are calculated by unpaired two-sided Student's t test. $*p < 0.05$; $**p < 0.01$; and ns, $p > 0.05$.

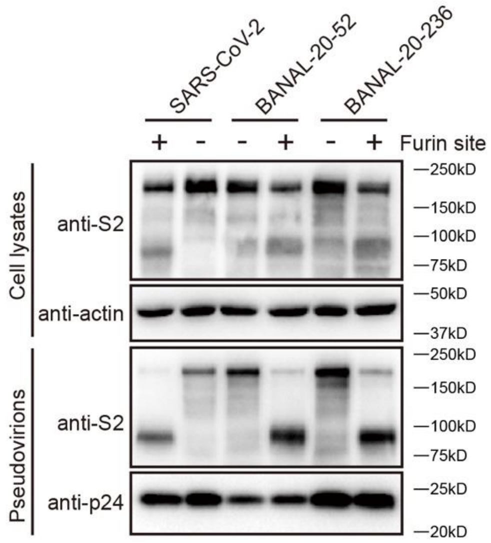

Supplementary Fig. S4 Western blotting of SARS-CoV-2 S, BANAL-20-52 S and BANAL-20-236 S proteins with or without a furin cleavage site. S proteins were detected using rabbit polyclonal anti-SARS-CoV-2 S2 antibody 40590-T62. β-actin and gag-p24 served as loading controls (cell lysates, top panel; pseudovirions, bottom panel).

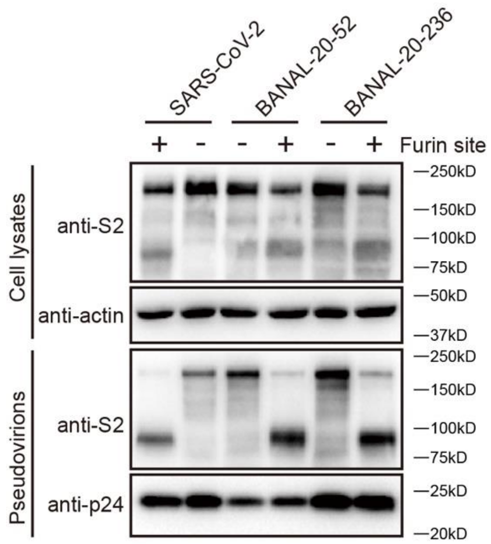

Supplementary Fig. S5 Western blot analysis of chimeric mutant BANAL-20-52 and BANAL-20-236 S proteins. S proteins were detected using rabbit polyclonal anti-SARS-CoV-2 S2 antibody 40590-T62. β-actin and gag-p24 served as loading controls (cell lysates, top panel; pseudovirions, bottom panel)

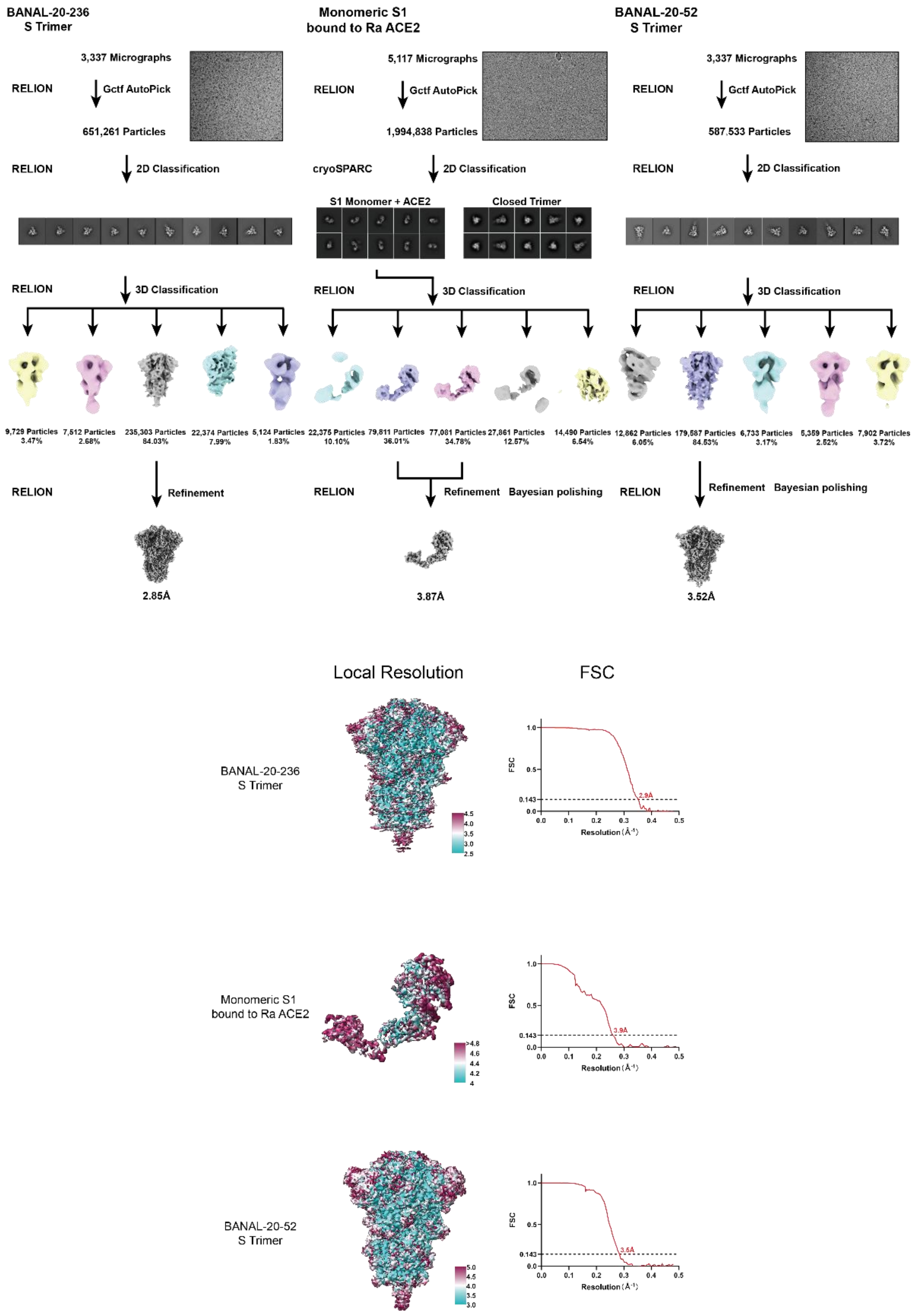

Supplementary Fig. S6 Processing schemes for cryo-EM data processing, and Fourier Shell Correlations (FSCs) and local resolution estimates for calculated maps.

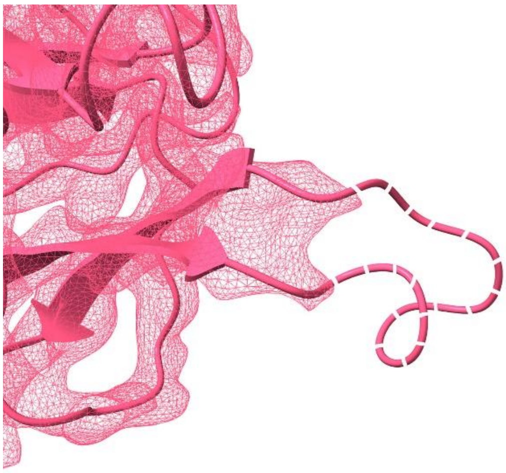

Supplementary Fig. S7 The structural and electronic map close to the furin site of BANAL-20-236 (PDBID: 8I3W). Dashed line indicates the potential structure of the furin site.

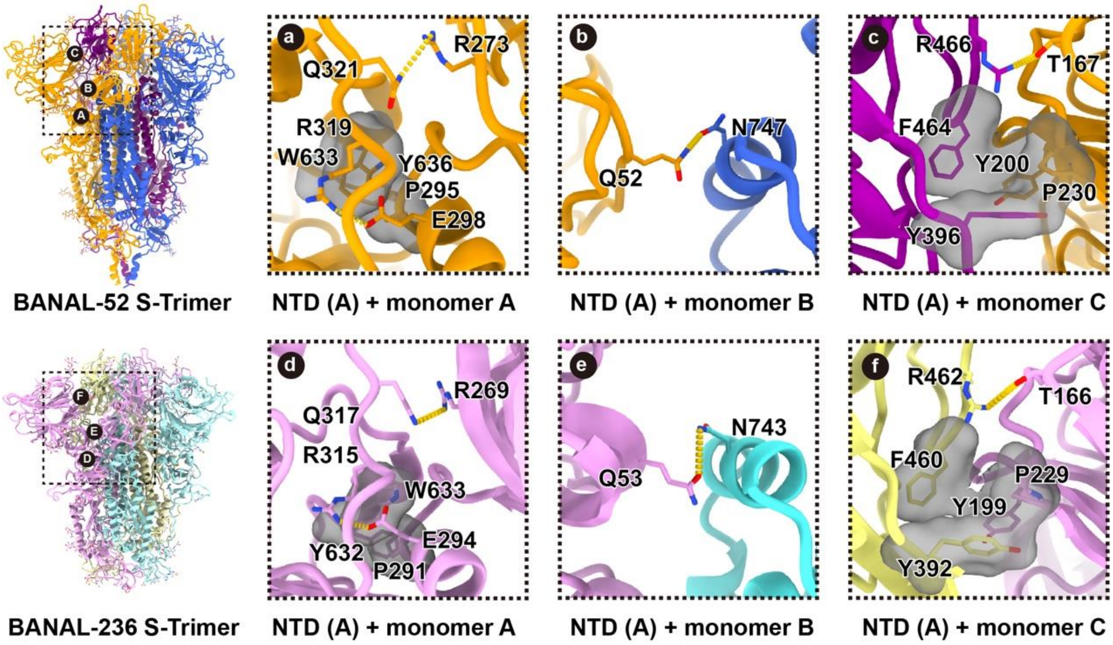

Supplementary Fig. S8 The representative interactions between NTDs with other regions of the S trimers of BANAL-20-52 (a, b, c) and BANAL-20-236 (d, e, f). While the individual monomers of BANAL-52 S trimer are colored as orange, blue, and purple, the individual monomers of BANAL-236 were colored as pink, cyan, and yellow, respectively. The hydrogen bonding and salt bridge are shown as the dashed yellow line, and the bulked electron cloud indicates the hydrophobic interactions.

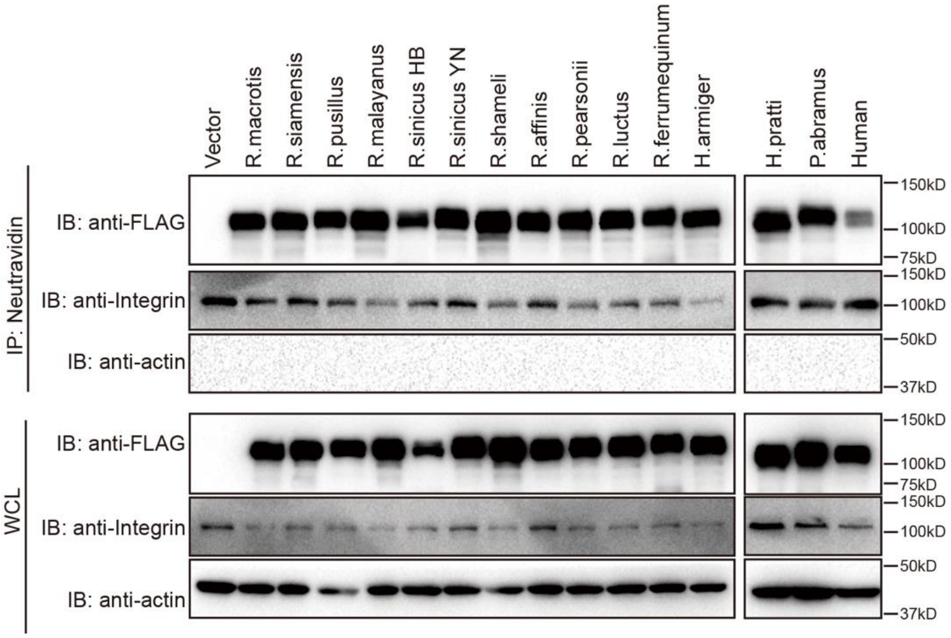

Supplementary Fig. S9 Cell surface expression of different bat ACE2 orthologs. HEK293 cells transiently overexpressing different bat species ACE2 proteins were labeled with EZ-link Sulfo-NHS-LC-LC-biotin on ice, and lysed with RIPA buffer. Biotinylated proteins were enriched with NeutrAvidin beads and analyzed by Western blotting using mouse monoclonal anti-FLAG M2 antibody. Integrin beta chain and β-actin were used as loading controls. WCL, whole cell lysate.

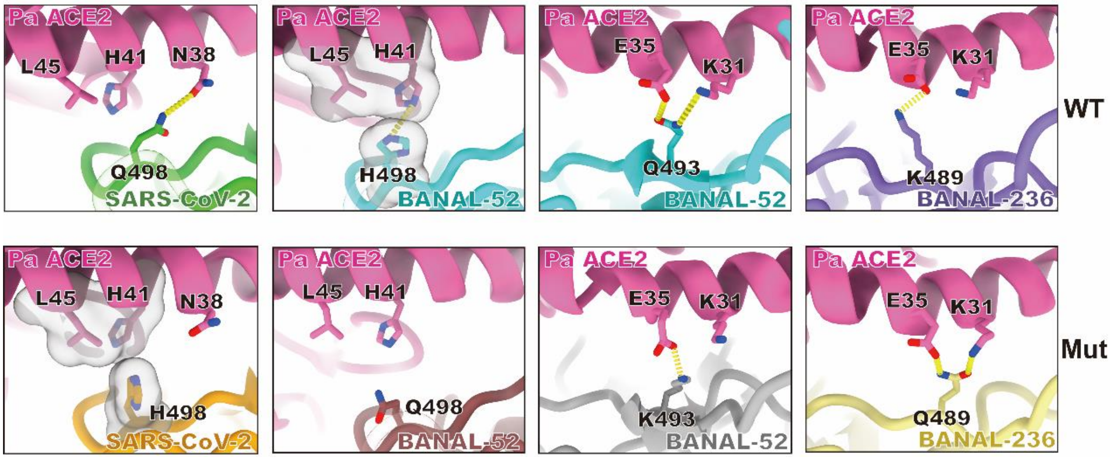

Supplementary Fig. S10 In silico analysis of interaction between *P. abramus* ACE2 and RBDs.

MD simulated interactions between *P. abramus* ACE2 and RBD of BANAL-20-236/52, SARS-CoV-2 mutants. Structures of the RBD and ACE2 are shown as ribbons.

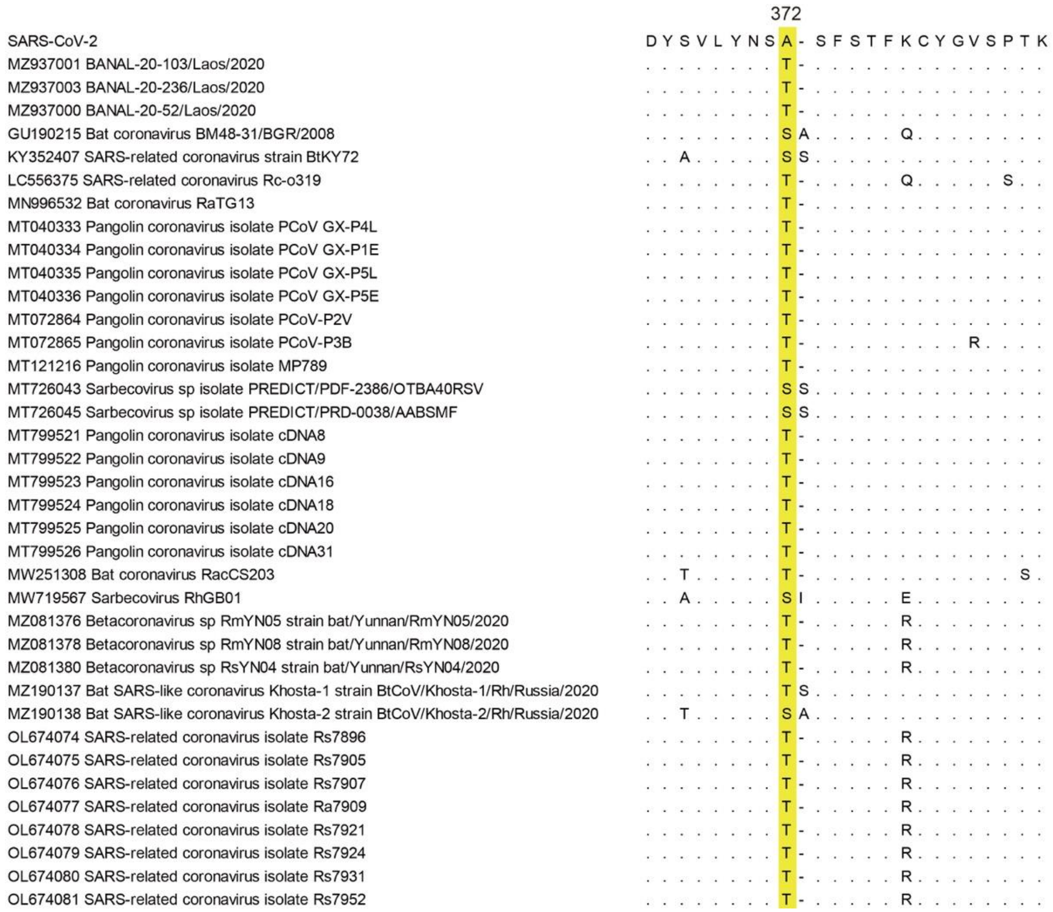

Supplementary Fig. S11 Alignment of the sequences around residue 372 of S proteins of bat SC2r-CoVs.

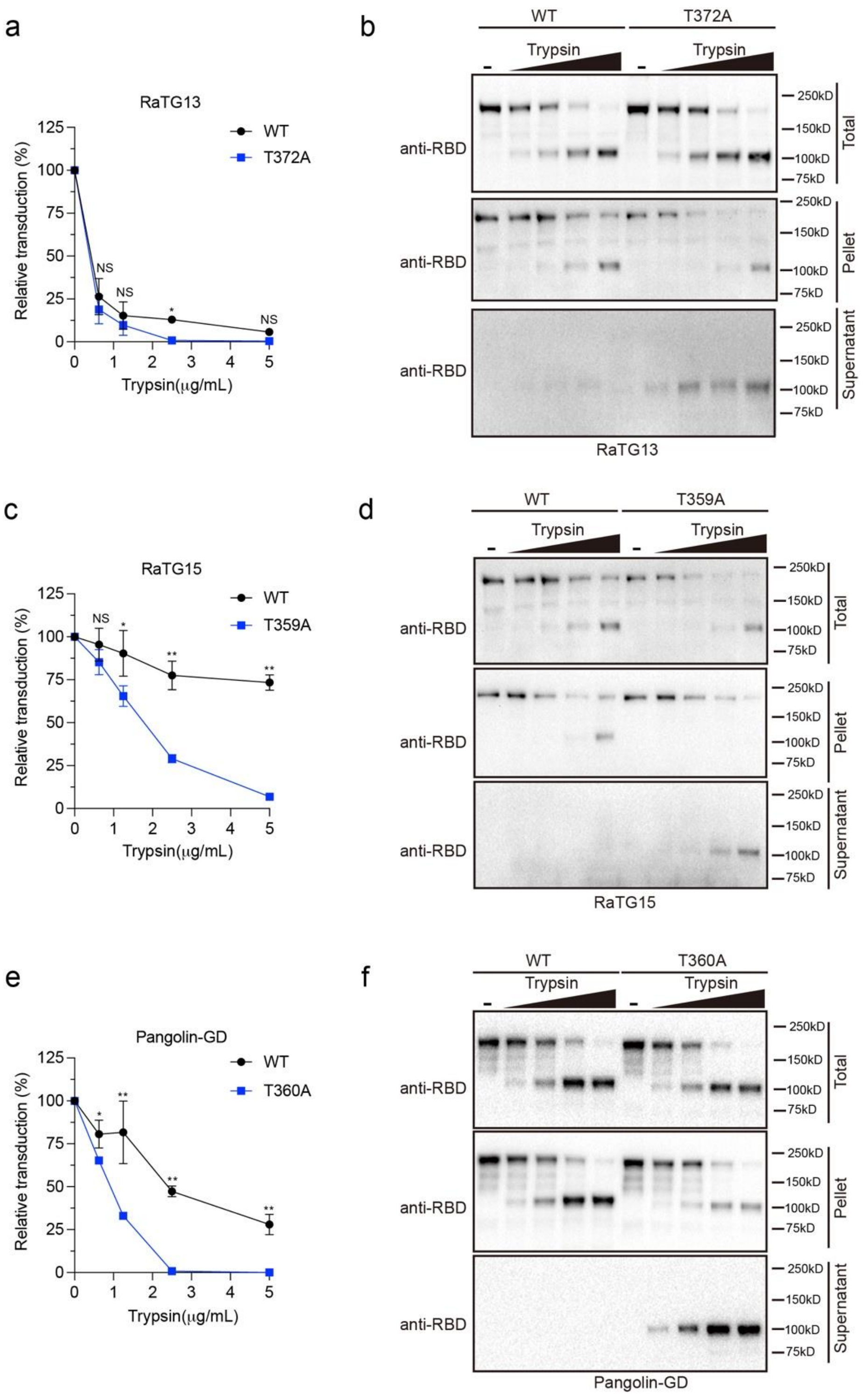

Supplementary Fig. S12 Proteolytic stability of SARS-CoV-2 like bat coronaviruses. (a, c, and e). Transduction of 293/hACE2 cells by SARS-CoV-2-like coronavirus RaTG13 S (a), RaTG15 S (c), Pangolin-GD S (e) wild-type and mutant pseudovirions. SARS-CoV-like S pseudovirions were first treated with serial dilutions of trypsin (2-fold dilution starting at 5μg/mL) at pH 5.5 for 10 min. After neutralization with trypsin inhibitor, pseudovirions were used to transduce 293/hACE2 at pH 7.4. (b, d, and f). Detection of S proteins in RaTG13 S (b), RaTG15 S (d), Pangolin-GD S (f) wild-type and mutant pseudovirions-pretreated with serial

dilutions of trypsin (2-fold dilution starting at 5μg/mL) at pH 5.5 for 10 min by Western blot analysis. Trypsin-treated pseudovirions were first centrifugated to separate the supernatants and pellets, and the S1 subunit in total, supernatants and pellets of pseudovirions were then detected by Western blot analysis using rabbit polyclonal anti-SARS-CoV-2 Spike RBD antibody.

Data are represented as mean ± standard deviation (SD) from at least triplicates. p values in (a, c and e) are calculated by unpaired two-sided Student's t test. *$p < 0.05$; **$p < 0.01$; and ns, $p > 0.05$.

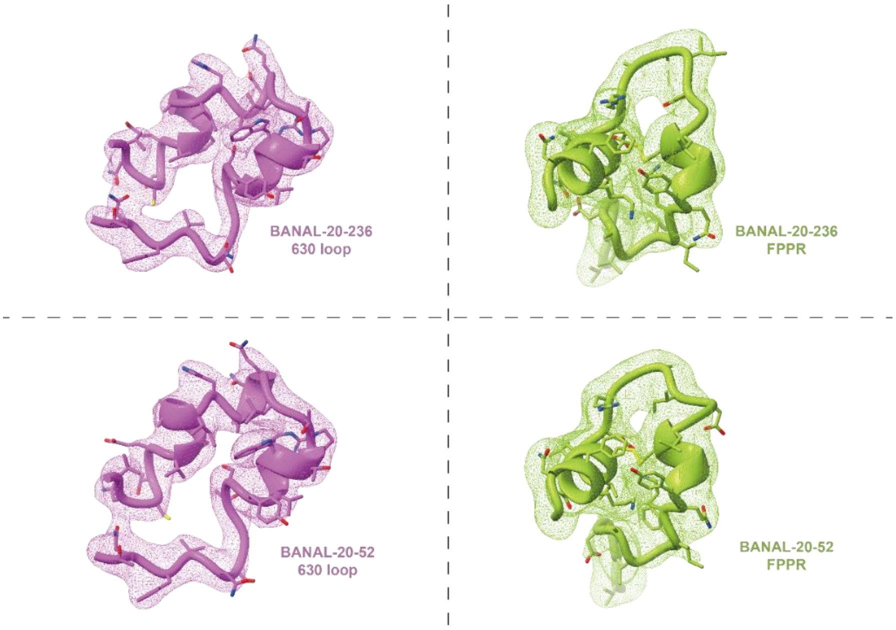

Supplementary Fig. S13 The EM map density of 630 loop and FPPR in BANAL-20-23 and BANAL-20-52 Spike. The atomic structures of 630 loop and FPPR are shown in magenta and green, respectively. The EM map densities of 630 loop and FPPR are represented by magenta and green meshes.

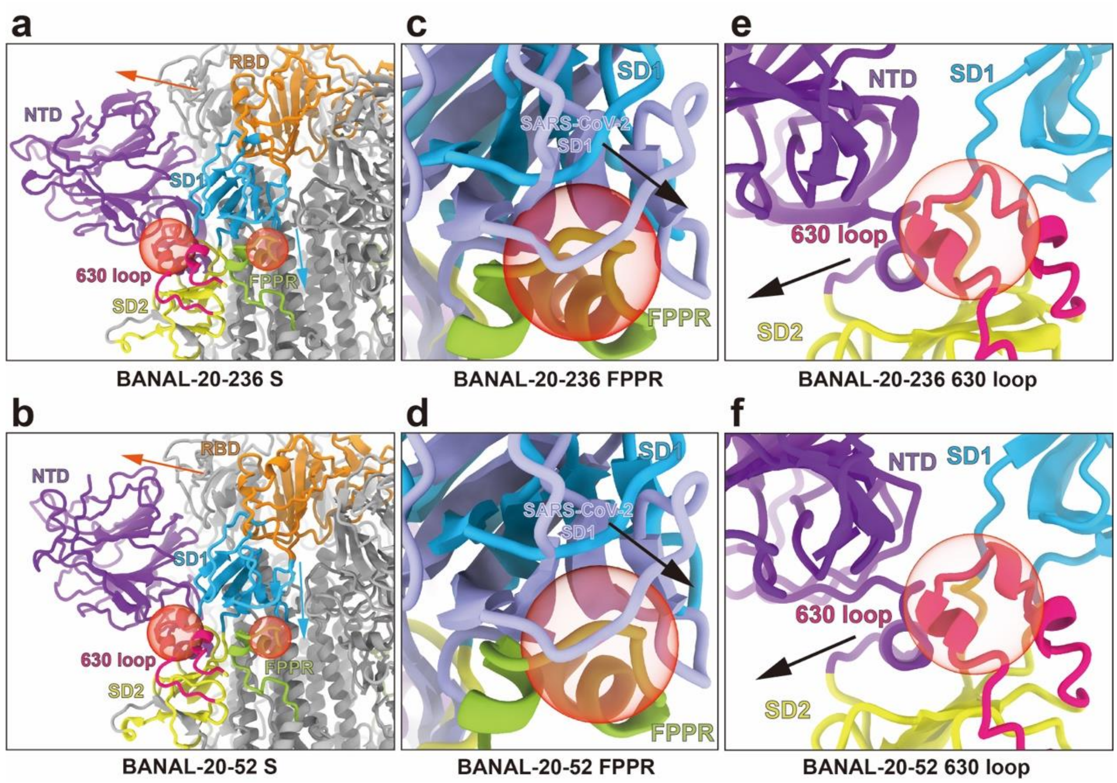

Supplementary Fig. S14 Effect of 630 Loop and FPPR on conformation of BANAL-20-236 and BANAL-20-52 S proteins. The RBDs of the BANAL-20-236 and BANAL-20-52 Spike are colored in orange, the SD1s are colored in blue, the NTD regions are colored in purple, the SD2 regions are colored in yellow, the FPPRs are colored in green, the 630 loops are colored in red and the red transparent sphere indicates where steric-hindrance effect might occur.

(a and b) The orange arrow indicates the change in the relative position of the raised RBD in the SARS-CoV-2 wild-type Spike trimer (PDB ID:6VYB) relative to RBDs of the two bat coronaviruses, and the blue arrow indicates the change in the relative position of SD1.

(c and d) The SD1 of SARS-CoV-2 one up formed Spike (PDB ID: 6VYB), which is superposed to the two bat coronavirus Spike, is colored orchid. The black arrow shows the relative position change of SARS-CoV-2 SD1 and two bat coronaviruses SD1.

(e and f) The black arrow indicates the relative position change of the 630 Loop of SARS-CoV-2 S protein (PDB ID: 6VYB) and the 630 loop of the two bat coronaviruses.

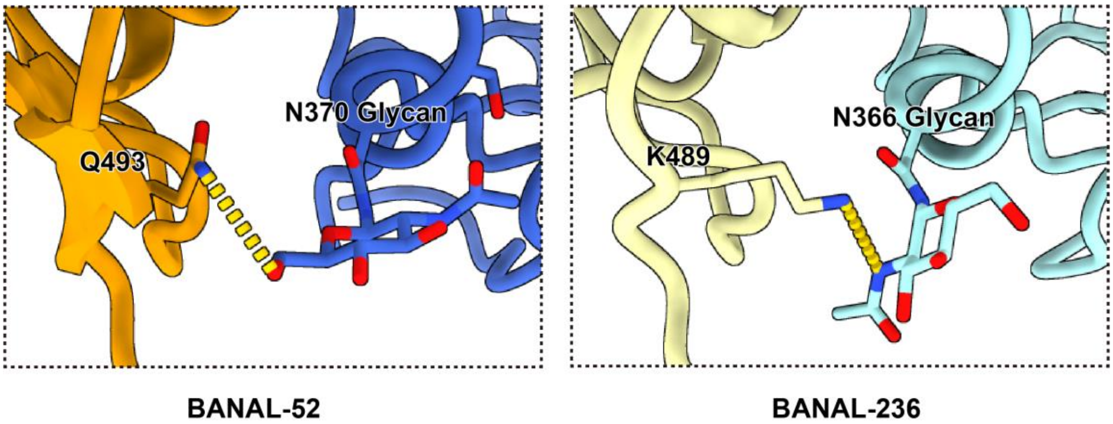

Supplementary Fig. S15 Detailed interaction between N370 glycan and Q493 from the adjacent monomer.

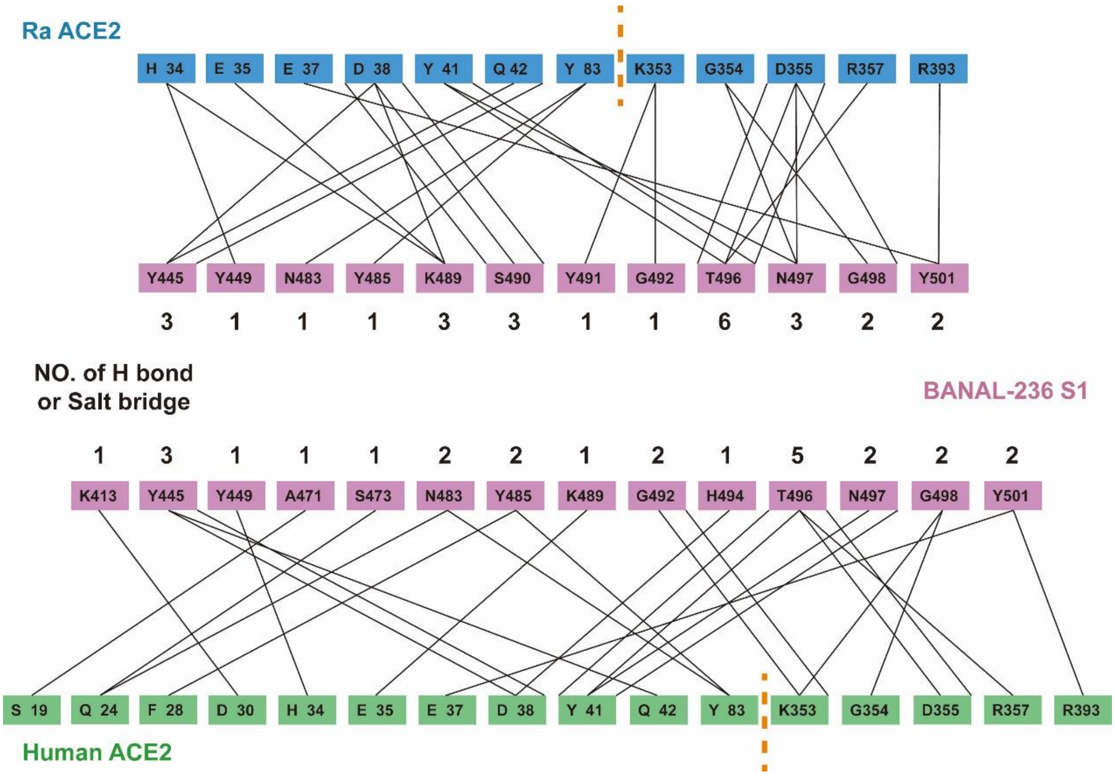

Supplementary Fig. S16 Comparison of hydrogen bond and salt bridge interaction networks between BANAL-20-236 S1/*R. affinis* ACE2 complex and BANAL-20-236 RBD /hACE2 complex. The purple boxes indicate the amino acid residues of BANAL-20-236 S1, the blue boxes indicate the amino acid residues of *R. affinis* ACE2, and the green boxes indicate the amino acid residues of human ACE2. The solid black and red lines show hydrogen bonds and salt bridges formed between indicated amino acid residues. The numbers in bold black below or above purple boxes indicate the number of hydrogen bonds and salt bridges formed by the corresponding amino acid residues. The orange dashed line divides the amino acid residues of ACE2, and the residues at both ends of the dashed line belong to different secondary structures.

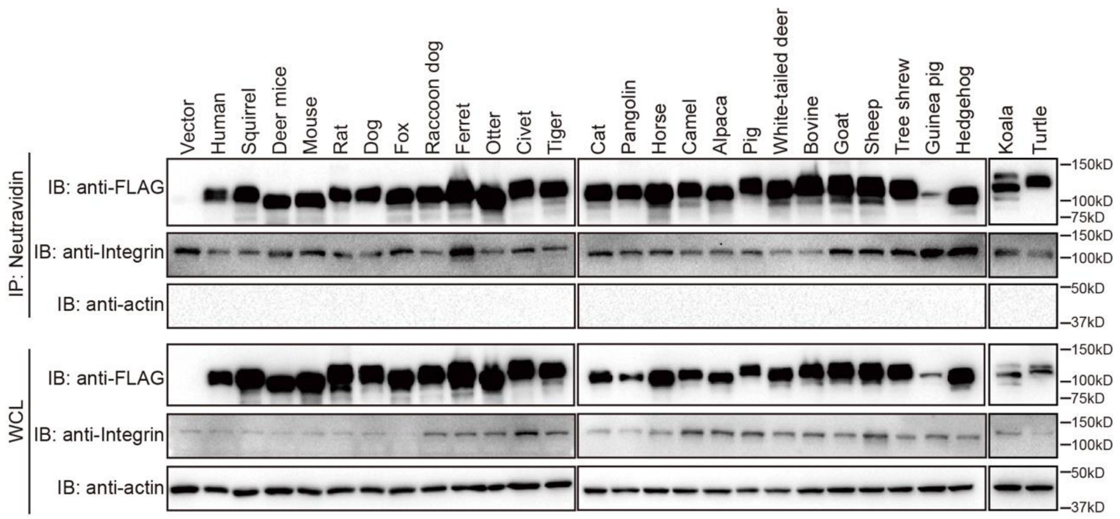

Supplementary Fig. S17 Cell surface expression of different bat ACE2 orthologs. Detection of different animal ACE2 proteins on cell surface by cell surface protein biotinylation assay. HEK293 cells transiently overexpressing different bat species ACE2 proteins were labeled with EZ-link Sulfo-NHS-LC-LC-biotin on ice, and lysed with RIPA buffer. Biotinylated proteins were enriched with NeutrAvidin beads and analyzed by Western blotting using mouse monoclonal anti-FLAG M2 antibody. Integrin beta chain and β-actin were used as loading controls. WCL, whole cell lysate.

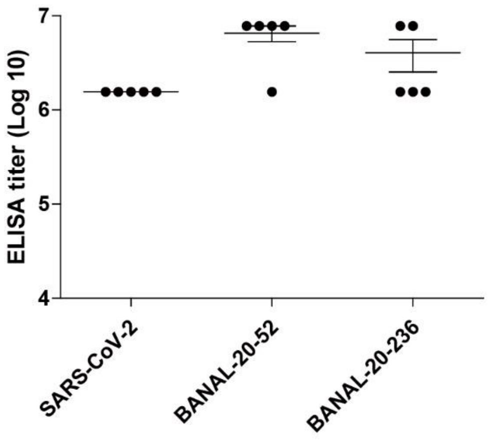

Supplementary Fig. S18 Detection of RBD-specific IgG antibodies of sera from S trimer-immunized mice by ELISA. Sera from SARS-CoV-2 S trimer, BANAL-20-52 S trimer or BANAL-20-236 S trimer-immunized mice were evaluated for their respective RBD-specific IgG antibodies by ELISA.

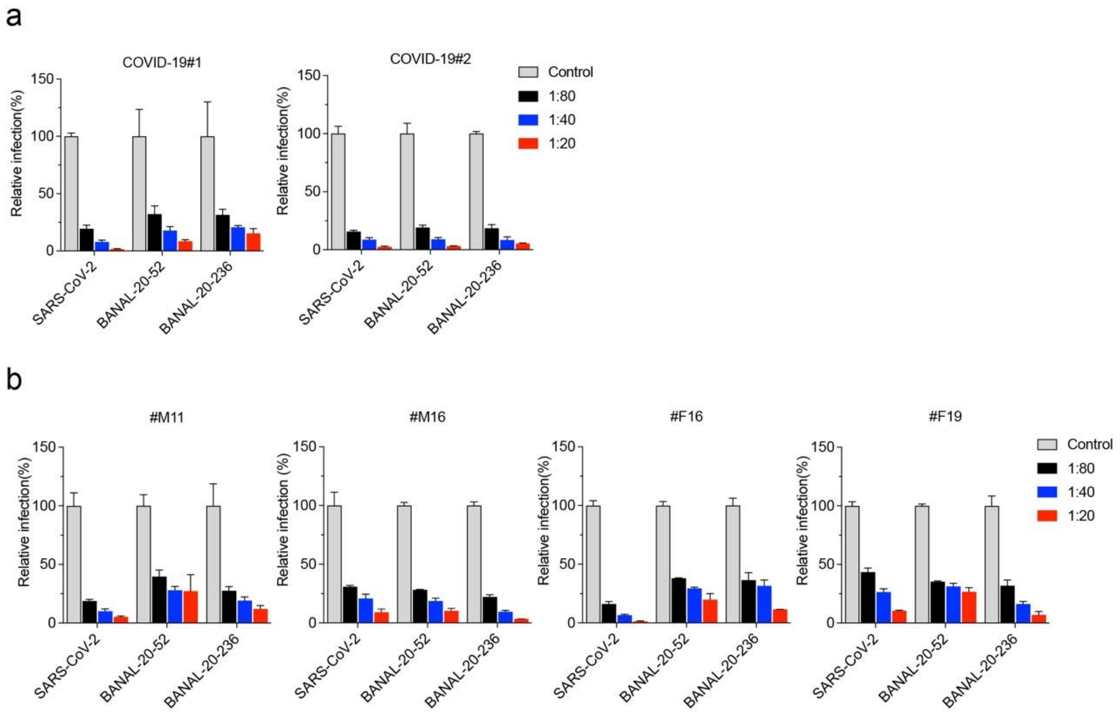

Supplementary Fig. S19 Cross-neutralization activities of human sera against BANAL-20-52 S and BANAL-20-236 S pseudovirions. (a and b). Neutralization titers of sera from recovered COVID-19 patients (a) and COVID-19 vaccine recipients (b) against SARS-CoV-2 S, BANAL-20-52 S, and BANAL-20-236 S pseudovirions.

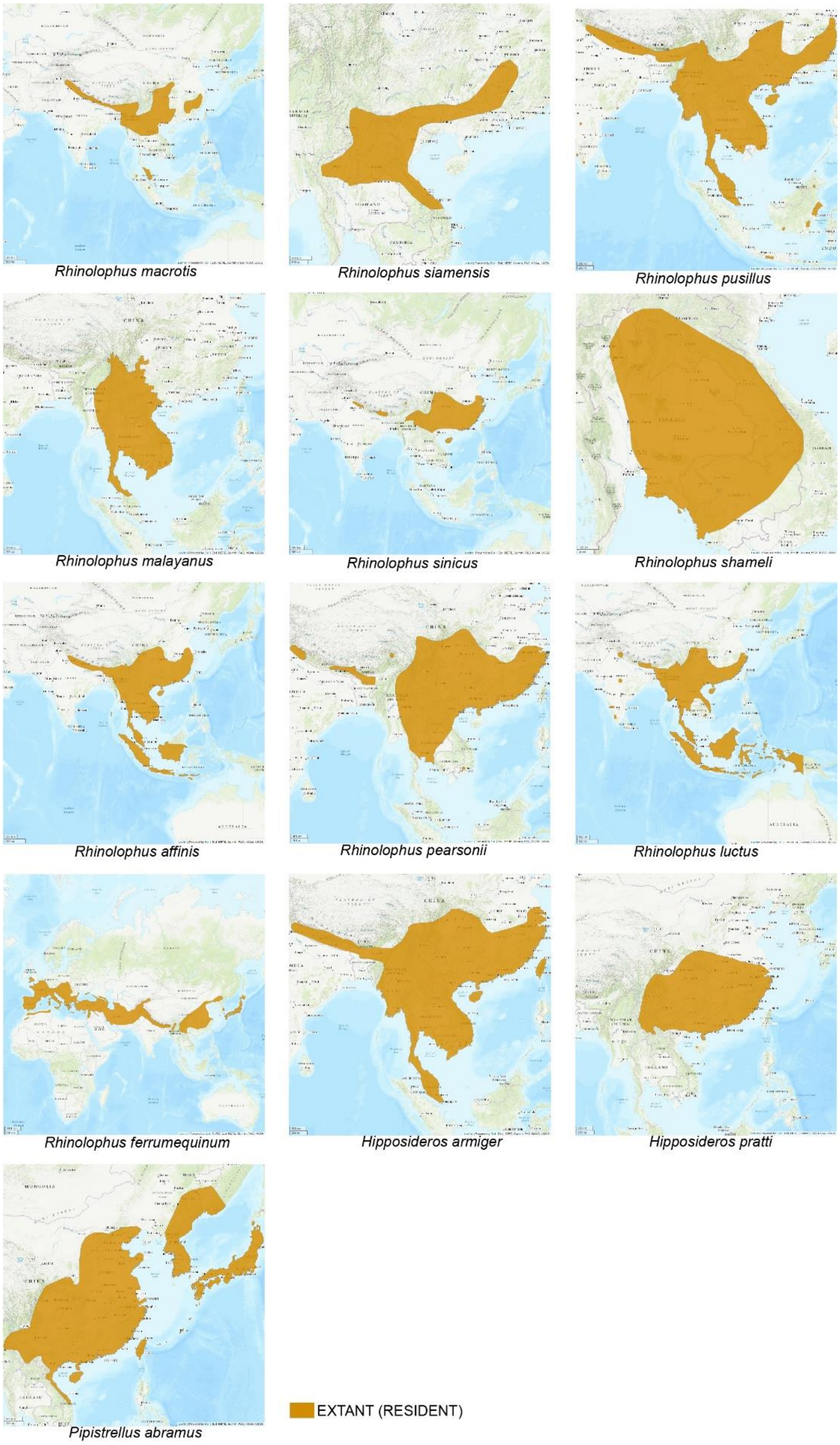

Supplementary Fig. S20 Geographic distribution of 13 bat species. (https://www.iucnredlist.org/)

Supplementary Table S1. The detailed interactions for BANAL-52 NTD (distance < 4 Å)

NTD of Chain A	Chain A	Chain B	Chain C
K41			L518
F43			F562, Q563
L50		L750	
Q52		T743, S746, N747, L750	
K113			S469
Q115			I468
E132			I468
N165			I468
N165 Glycan			Y351
T167			R466
D198			P463, F464
G199			P463, F464
Y200			F464, R355, Y396
P230			R355, Y396
I231			R466
G232			E465, F464, R466
N234			E465
N234 Glycan			E465, R457, S459, K468
R273	Q321		
N282			L560
C291	F318		
S292	F318, T630, W633		
L293	T630, T632, W633		
D294	T632, W633		
P295	V597, V610, W633, Y636		
E298	T315, S316, F318, R319 W633		
T299	T315, Y313, V597		
C301	F318		
T302	T315, S316		
L303	Y313		
K304		T757	

Supplementary Table S2. The detailed interactions for BANAL-236 NTD (distance < 4 Å)

NTD of Chain A	Chain A	Chain B	Chain C
T42			L514
F44			N515, Q559
L51		L746	
Q53		T739, S742, N743, L746	
T113			S465, E467
Q115			I464
T166			R462
D197			P459, F460
G198			P459, F460
Y199			R351, Y392, F460
P229			R351, Y392
G231			F460, E461, R462
N233 Glycan			R453, S455, K454, K458, E461
P268	Q317		
R269	Q317		
T270	F314		
N278			L556
C287	F314		
S288	F314, Q317, T626, W629		
L289	T626, T628, W629		
D290	T628, W629,		
P291	V593, V604, V606, W629, Y632		
L292	T595, N602, V604		
E294	T311, F314, R315, W629		
A295	T311		
K296	T598		
C297	F314		
T298	Q310, T311, S312		
K300		S750	

Supplementary Table S3. The detailed interactions between the NTD and neighbouring domains of the S protein

S52-NTD (chain A)				S236-NTD (chain A)			
self: 34 contacts		cross: 37 contacts		cross: 30 contacts		self: 31 contacts	
S52:chain B	S52:chain C	S236:chain B	S236:chain C	S52:chain B	S52:chain C	S236:chain B	S236:chain C
6	28	6	31	6	24	6	25
50L......L750	41K......L518	50L......L746	41K......L514	51L......L750	42T......E516	51L......L746	42T......L514
52Q......L750	43F......F562	52Q......L746	43F......N515	53Q......L750	42T......L518	53Q......L746	44F......N515
52Q......N747	43F......Q563	52Q......N743	43F......Q559	53Q......N747	44F......N519	53Q......N743	44F......Q559
52Q......S746	113K......S469	52Q......S742	113K......E467	53Q......S746	44F......Q563	53Q......S742	113T......E467
52Q......T743	115Q......I468	52Q......T739	113K......S465	53Q......T743	44F......R567	53Q......T739	113T......S465
304K......T757	132E......I468	304K......T753	115Q......I464	300K......S754	115Q......I468	300K......S750	115Q......I464
	165N......I468		132E......S465		166T......R466		166T......R462
	167T......R466		198D......F460		197D......F464		197D......F460
	198D......F464		198D......P422		197D......P463		197D......P459
	198D......P463		198D......P459		198G......F464		198G......F460
	199G......F464		199G......E461		198G......P463		198G......P459
	199G......P463		199G......F460		199Y......N394		199Y......R351
	200Y......R355		199G......P459		199Y......R355		199Y......Y392
	200Y......Y396		200Y......F460		199Y......Y396		199Y......F460
	200Y......F464		200Y......R351		229P......R355		229P......R351
	230P......R355		200Y......Y392		229P......Y396		229P......Y392
	230P......Y396		230P......R351		231G......E465		231G......E461
	231I......R466		230P......Y392		231G......F464		231G......F460
	232G......E465		231I......R462		231G......R466		231G......R462
	232G......F464		232G......E461		278N......L560		278N......L556

	232G......R466		232G......F460		233N glycan…E465		233N glycan…R453
	234N......E465		232G......R462		233N glycan…R457		233N glycan…S455
	282N......L560		233I......E461		233N glycan…S459		233N glycan…K454
	165N glycan…Y351		233I......R462		233N glycan…K468		233N glycan…K458
	234N glycan…E465		234N......E461				233N glycan…E461
	234N glycan…R457		282N......L556				
	234N glycan…S459		234N glycan…R453				
	234N glycan…K468		234N glycan…S455				
			234N glycan…K454				
			234N glycan…K458				
			234N glycan…E461				

Supplementary Table S4. Accession number list of bat and animal ACE2s

Bat species	Accession
Rhinolophus macrotis	ADN93471.1
Rhinolophus siamensis	*Ref
Rhinolophus pusillus	ADN93477.1
Rhinolophus malayanus	*Ref
Rhinolophus sinicus HB	ADN93475
Rhinolophus sinicus YN	AGZ48803
Rhinolophus shameli	UBB59645
Rhinolophus affinis	QMQ39242.1
Rhinolophus pearsonii	QKE49996.1
Rhinolophus luctus	*Ref
Rhinolophus ferrumequinum	XP_032963186.1
Hipposideros armiger	XP_019522954.1
Hipposideros pratti	QKE49995.1
Rhinolophus macrotis	ADN93471.1
**Animal species**	**Accession**
Squirrel	XP_026252505
Deer mice	XP_006973269.1
Mouse	NP_001123985
Rat	NP_001012006
Dog	NP_001158732.1
Fox	XP_025842512
Raccoon dog	ABW16956.1
Ferret	NP_001297119
Otter	XP_032736029.1
Civet	AAX63775
Tigers	XP_042830021.1
Cat	AAX59005.1
Pangolin	XP_017505746
Horse	XP_001490241.1
Camel	XP_006194263
Alpaca	XP_006212709.1
Pig	NP_001116542
White-tailed deer	XP_020768965.1
Bovine	NP_001019673.2
Goat	NP_001277036.1
Sheep	XP_011961657.1

Tree shrews	QNV47311.1
Guinea pig	ACT66270
Hedgehog	XP_004710002
Koala	XP_020863153
Turtle	XP_006122891

*Ref: The ACE sequences of *Rhinolophus siamensis, Rhinolophus malayanus,* and *Rhinolophus luctus* were obtained from the previous study (Wu et al., National Science Review, nwac213, 2022).

Supplementary Table S5. Cryo-EM data Collection, refinement and validation statistic

Protein	BANAL-20-236 S-trimer	BANAL-20-52 S-trimer
**Data collection**		
Voltage (kV)	300	300
Microscope	FEI Titan Krios G2	FEI Titan Krios G2
Camera	K2 (Gatan)	K2 (Gatan)
Magnification (calibrated)	130,000X	130,000X
Electron exposure (e^{-}/Å^2)	60	60
Frames rate	32	32
Defocus range (μm)	-1.4 to -2.2	-1.4 to -2.2
Pixel size (Å)	1.04	1.04
**Overall map processing**		
Micrographs used	3,337	3125
Symmetry imposed	C3	C3
Initial particle images	651,261	587,533
Final particle images	235,303	179,587
Resolution at 0.143 FSC of masked reconstruction (Å)	2.85	3.52
Map sharpening B factor (Å^2)	-102.8	-132.0
**Local map refinement**		
Initial model used (PDB code)	6VXX	6VXX
Refinement package	Phenix v1.19	Phenix v1.19
**Model composition**		
Non-hydrogen atoms	27,000	27,639
Protein residues	3345	3399
Ligands	NAG:69	NAG:81

Supplementary Table S6. Cryo-EM data Collection, refinement and validation statistic (continued)

Protein	BANAL-20-236 S-trimer	BANAL-20-52 S-trimer
**R.m.s. deviations**		
Bond lengths (Å)	0.004	0.003
Bond angles (°)	0.671	0.619
**B factors ($Å^2$)**		
Protein	32.42	77.42
Ligands	49.18	77.07
**Validation**		
MolProbity score	2.11	2.12
Clashscore	12.97	11.90
Poor rotamers (%)	0.00	0.00
**Ramachandran plot**		
Favored (%)	92.00	90.60
Allowed (%)	8.00	9.40
Disallowed (%)	0.00	0.00
Cβ outliers (%)	0.00	0.00
CaBLAM outliers (%)	6.03	6.85

Supplementary Table S7. Cryo-EM data Collection, refinement and validation statistic

Protein	BANAL-20-236 S1 and *R. affinis* ACE2
**Data collection**	
Voltage (kV)	300
Microscope	FEI Titan Krios
Camera	K3 (Gatan)
Magnification (calibrated)	22,500X
Electron exposure ($e^{-}/Å^2$)	60
Frames rate	32
Defocus range (μm)	-1.4 to -2.2
Pixel size (Å)	1.07
**Overall map processing**	
Micrographs used	5,117
Symmetry imposed	C1
Initial particle images	1,994,838
Final particle images	156,892
Resolution at 0.143 FSC of masked reconstruction (Å)	3.87
Map sharpening B factor ($Å^2$)	-128.6
**Local map refinement**	
Initial model used (PDB code)	7R10
Refinement package	Phenix v1.19
**Model composition**	
Non-hydrogen atoms	10,340
Protein residues	1273
Ligands	NAG:9

Supplementary Table S8. Cryo-EM data Collection, refinement and validation statistic (continued)

Protein	BANAL-20-236 S1 and *R. affinis* ACE2
**R.m.s. deviations**	
Bond lengths (Å)	0.006
Bond angles (°)	1.269
**B factors (Å^2)**	
Protein	134.8
Ligands	118.34
**Validation**	
MolProbity score	2.15
Clashscore	13.00
Poor rotamers (%)	0.00
**Ramachandran plot**	
Favored (%)	90.78
Allowed (%)	9.22
Disallowed (%)	0.00
Cβ outliers (%)	0.00
CaBLAM outliers (%)	5.69

KEY RESOURCES TABLE

REAGENT OR RESOURCE	SOURCE	IDENTIFIER
Antibodies		
Mouse monoclonal anti-FLAG M2 antibody	Sigma	Cat: F1804-5MG
Mouse monoclonal anti-β-actin antibody	Sigma	Cat: A5441-2ML
Rabbit polyclonal anti-HIV-1 Gag-p24 antibody	Sino Biological Inc. (Beijing, China)	Cat: 11695-RP01
Rabbit polyclonal anti-SARS-CoV-2 S2 antibody	Sino Biological Inc. (Beijing, China)	Cat: 40590-T62
Rabbit polyclonal anti-SARS-CoV-2 Spike RBD antibody	Sino Biological Inc. (Beijing, China)	Cat:40592-T62
Rabbit polyclonal anti-integrin β1 antibody	Proteintech (Wuhan, China)	Cat: 34971
Mouse anti-strep-tag II antibody	Bioss Biotechnology, (Beijing, China)	Cat: bsm-33016M
HRP-conjugated donkey anti-rabbit IgG	Jackson ImmunoResearch (West Grove, PA, USA)	Cat: 711-035-152
HRP-conjugated goat anti-mouse IgG	Jackson ImmunoResearch (West Grove, PA, USA)	Cat: 115-035-146
Bacterial and virus strains		
Trelief 5α chemically competent Cell	TSINGKE Biotechnology Co., Ltd	Cat: TSC-C01
Biological samples		
Human COVID-19 convalescent plasmas	(Ou et al., 2020)	N/A
Sera from persons immunized with inactivated SARS-CoV-2 vaccine	This study	N/A
Sera from recombinant spike-immunized mice	This study	N/A
Chemicals, peptides, and recombinant proteins		
E64D	Med Chem Express (New Jersey, USA)	Cat: HY-100229
Bafilomycin A1	Med Chem Express (New Jersey, USA)	Cat: HY-100558
Apilimod	Med Chem Express (New Jersey, USA)	Cat: HY-14644
Camostat	Selleck Chemicals (Texas, USA)	Cat: S2874
EZ-linked Sulfo-NHS-LC-LC-biotin	Thermo Pierce	Cat: #21331
SARS-CoV-2 S-trimer	Sino Biological Inc. (Beijing, China)	Cat: 40589-V08H4
BANAL-20-52 S-trimer (K982P/V983P)	This study	N/A
BANAL-20-236 S-trimer (K978P/V979P)	This study	N/A
Soluble *R. affinis* ACE2	This study	N/A
SARS-CoV-2 RBD	This study	N/A
BANAL-20-52 RBD	This study	N/A

BANAL-20-236 RBD	This study	N/A
Critical commercial assays		
Steady-Glo® Luciferase Assay System	Invitrogen	Cat: E2510
Experimental models: Cell lines		
HEK293T	ATCC	Cat: CRL-3216
HEK293	ATCC	Cat: CRL-1573
HEK293/hACE2	Laboratory of Zhaohui Qian	N/A
Calu3	ATCC	Cat: HTB-55
Expi293F	Gibco	Cat: A14527
Experimental models: Organisms/strains		
BALB/c mice	Beijing Vital River Laboratory Animal Technology Co., Ltd	N/A
Oligonucleotides		
SARS-CoV-2-S-Q498H-F: CTACGGCTTCCACCCAACCAACGGC	Ruibo Xingke Biotech (Beijing, China)	N/A
SARS-CoV-2-S-Q498H-R: GCCGTTGGTTGGGTGGAAGCCGTAG	Ruibo Xingke Biotech (Beijing, China)	N/A
BANAL-20-52-S-T372A-F: CTGTGCTGTATAACAGCGCCTCTTTCAGCACATTCAAG	Ruibo Xingke Biotech (Beijing, China)	N/A
BANAL-20-52-S-T372A-R: CTTGAATGTGCTGAAAGAGGCGCTGTTATACAGCACAG	Ruibo Xingke Biotech (Beijing, China)	N/A
BANAL-20-52-S-Q493K-F: GTTACTTTCCTCTGAAAAGCTACGGCTTCC	Ruibo Xingke Biotech (Beijing, China)	N/A
BANAL-20-52-S-Q493K-R: GGAAGCCGTAGCTTTTCAGAGGAAAGTAAC	Ruibo Xingke Biotech (Beijing, China)	N/A
BANAL-20-52-S-H498Q-F: CTACGGCTTCCAACCTACCAACGGC	Ruibo Xingke Biotech (Beijing, China)	N/A
BANAL-20-52-S-H498Q-R: GCCGTTGGTAGGTTGGAAGCCGTAG	Ruibo Xingke Biotech (Beijing, China)	N/A
BANAL-20-236-S-T368A-F: CAGCGTGCTTTATAACAGCGCCAGCTTCAGCACATTTAAG	Ruibo Xingke Biotech (Beijing, China)	N/A
BANAL-20-236-S-T368A-R: CTTAAATGTGCTGAAGCTGGCGCTGTTATAAAGCACGCTG	Ruibo Xingke Biotech (Beijing, China)	N/A
BANAL-20-236-S-K489Q-F: GCTACTTCCCCCTGCAGAGCTACGGCTTTC	Ruibo Xingke Biotech (Beijing, China)	N/A

BANAL-20-236-S-K489Q-R: GAAAGCCGTAGCTCTGCAGGGGGAAGTAGC	Ruibo Xingke Biotech (Beijing, China)	N/A
BANAL-20-52-S-2P(K982P/V983P)-F: CTGAGCAGACTGGATCCCCCTGAGGCCGAGGTGCAG	Ruibo Xingke Biotech (Beijing, China)	N/A
BANAL-20-52-S-2P(K982P/V983P)-R: CTGCACCTCGGCCTCAGGGGGATCCAGTCTGCTCAG	Ruibo Xingke Biotech (Beijing, China)	N/A
BANAL-20-236-S-2P(K978P/V979P)-F: CCTGAGCAGACTGGATCCACCCGAAGCCGAGGTGCAG	Ruibo Xingke Biotech (Beijing, China)	N/A
BANAL-20-236-S-2P(K978P/V979P)-R: CTGCACCTCGGCTTCGGGTGGATCCAGTCTGCTCAGG	Ruibo Xingke Biotech (Beijing, China)	N/A
BANAL-20-52-S-ins-furin-F: CTGCACCTCGGCTTCGGGTGGATCCAGTCTGCTCAGG	Ruibo Xingke Biotech (Beijing, China)	N/A
BANAL-20-52-S-ins-furin-R: GCGATGATGCTCTGAGATGCTCTCCTTGGGCTGTTGGTTTGGGTCTG	Ruibo Xingke Biotech (Beijing, China)	N/A
BANAL-20-236-S-ins-furin-F: CAGACACAGACCAATTCCCCAAGGAGAGCAAGATCCGTGGCCAGCCAG	Ruibo Xingke Biotech (Beijing, China)	N/A
BANAL-20-236-S-ins-furin-R: CTGGCTGGCCACGGATCTTGCTCTCCTTGGGGAATTGGTCTGTGTCTG	Ruibo Xingke Biotech (Beijing, China)	N/A
SARS-CoV-2-S-del-furin-F: CCAGACAAACTCCCCACGGTCTGTGGCAAGC	Ruibo Xingke Biotech (Beijing, China)	N/A
SARS-CoV-2-S-del-furin-R: GCTTGCCACAGACCGTGGGGAGTTTGTCTGG	Ruibo Xingke Biotech (Beijing, China)	N/A
Pangolin-GD-S-T368A-F: CGTGCTGTACAACAGCGCCAGCTTCAGCACATTC	Ruibo Xingke Biotech (Beijing, China)	N/A
Pangolin-GD-S-T368A-R: GAATGTGCTGAAGCTGGCGCTGTTGTACAGCACG	Ruibo Xingke Biotech (Beijing, China)	N/A
RaTG15-S-T359A-F: GCTCTACAACAGCGCCTCTTTCAGTACC	Ruibo Xingke Biotech (Beijing, China)	N/A
RaTG15-S-T359A-R: GGTACTGAAAGAGGCGCTGTTGTAGAGC	Ruibo Xingke Biotech (Beijing, China)	N/A

RaTG13-S-T372A-F: GCTGTATAATAGCGCCAGCTTCAGCACC	Ruibo Xingke Biotech (Beijing, China)	N/A
RaTG13-S-T372A-R: GGTGCTGAAGCTGGCGCTATTATACAGC	Ruibo Xingke Biotech (Beijing, China)	N/A
Recombinant DNA		
pcDNA3.1-SARS-CoV-S-delta19	Laboratory of Zhaohui Qian	N/A
p3xFLAG-CMV14-SARS-CoV-2-S-delta19	Laboratory of Zhaohui Qian	N/A
p3xFLAG-CMV14-BANAL-20-52-S-delta19	This study	GenBank: MZ937000
p3xFLAG-CMV14-BANAL-20-236-S-delta19	This study	GenBank: MZ937003
p3xFLAG-CMV14-Pangolin-GD-S-delta19	This study	GenBank: MT799521.1
p3xFLAG-CMV14-RaTG13-S-delta19	(Li et al.,2021)	GISAID accession number: EPI_ISL_6640919
p3xFLAG-CMV14-Omicron-S-delta19	This study	GISAID accession number: EPI_ISL_6640919
p3xFLAG-CMV14-Delta-S-delta19	This study	GISAID accession number: EPI_ISL_3940074
p3xFLAG-CMV14-RaTG15-S-delta19	This study	National Genomics Data Center of China accession number: GWHBAUP01000000
p3xFLAG-CMV14-SARS-CoV-2-S-Q498H	This study	N/A
p3xFLAG-CMV14- BANAL-20-52-S-T472A	This study	N/A
p3xFLAG-CMV14-BANAL-20-52-S-Q493K	This study	N/A
p3xFLAG-CMV14-BANAL-20-52-S-H498Q	This study	N/A
p3xFLAG-CMV14-BANAL-20-236-S-T368A	This study	N/A
p3xFLAG-CMV14-BANAL-20-236-S-K489Q	This study	N/A
p3xFLAG-CMV14-RaTG13-S-T372A	This study	N/A
p3xFLAG-CMV14-RaTG15-S-T359A	This study	N/A
p3xFLAG-CMV14-Pangolin-GD-S-T368A	This study	N/A
p3xFLAG-CMV14-SARS-CoV-2-S-Del-furin	This study	N/A
p3xFLAG-CMV14-BANAL-20-52-S-Ins-furin	This study	N/A
p3xFLAG-CMV14-BANAL-20-236-S-Ins-furin	This study	N/A
pcDNA3.1-BANAL-20-52-S-trimer (2P)	This study	N/A
pcDNA3.1-BANAL-20-236-S-trimer (2P)	This study	N/A
pcDNA3.1-SARS-CoV-2-RBD	This study	N/A
pcDNA3.1-BANAL-20-52-RBD	This study	N/A
pcDNA3.1-BANAL-20-236-RBD	This study	N/A
pcDNA3.1-sRaACE2	This study	N/A
psPAX2	Addgene	Cat: 12260
pLenti-GFP	A gift of Fang Li (Duke University)	N/A

p3xFLAG-CMV14-human ACE2, accession number: NP_068576	(Li et al., 2021)	N/A
p3xFLAG-CMV14-squirrel ACE2, accession number: XP_026252505	(Li et al., 2021)	N/A
p3xFLAG-CMV14-pangolin ACE2, accession number: XP_017505746	(Li et al., 2021)	N/A
p3xFLAG-CMV14-fox ACE2, accession number: XP_025842512	(Li et al., 2021)	N/A
p3xFLAG-CMV14-civet ACE2, accession number: AAX63775	(Li et al., 2021)	N/A
p3xFLAG-CMV14-camel ACE2, accession number: XP_006194263	(Li et al., 2021)	N/A
p3xFLAG-CMV14-ferret ACE2, accession number: NP_001297119	(Li et al., 2021)	N/A
p3xFLAG-CMV14-rat ACE2, accession number: NP_001012006	(Li et al., 2021)	N/A
p3xFLAG-CMV14-mouse ACE2, accession number: NP_001123985	(Li et al., 2021)	N/A
p3xFLAG-CMV14-pig ACE2, accession number: NP_001116542	(Li et al., 2021)	N/A
p3xFLAG-CMV14-guinea pig ACE2 accession number: ACT66270	(Li et al., 2021)	N/A
p3xFLAG-CMV14-deer ACE2 accession number: XP_020768965	(Li et al., 2021)	N/A
p3xFLAG-CMV14-hedgehog ACE2 accession number: XP_004710002	(Li et al., 2021)	N/A
p3xFLAG-CMV14-koala ACE2 accession number: XP_020863153	(Li et al., 2021)	N/A
p3xFLAG-CMV14-turtle ACE2 accession number: XP_006122891	(Li et al., 2021)	N/A
p3xFLAG-CMV14-deer mice ACE2, accession number: XP_006973269.1	This study	N/A
p3xFLAG-CMV14-dog ACE2, accession number: NP_001158732.1	This study	N/A
p3xFLAG-CMV14-raccoon dog ACE2, accession number: ABW16956.1	This study	N/A
p3xFLAG-CMV14-otter ACE2, accession number: XP_032736029.1	This study	N/A
p3xFLAG-CMV14-tiger ACE2, accession number: XP_042830021.1	This study	N/A
p3xFLAG-CMV14-cat ACE2, accession number: AAX59005.1	This study	N/A
p3xFLAG-CMV14-horse ACE2, accession number: XP_001490241.1	This study	N/A

p3xFLAG-CMV14-alpaca ACE2, accession number: XP_006212709.1	This study	N/A
p3xFLAG-CMV14-white-tailed deer ACE2, accession number: XP_020768965.1	This study	N/A
p3xFLAG-CMV14-bovine ACE2, accession number: NP_001019673.2	This study	N/A
p3xFLAG-CMV14-goat ACE2, accession number: NP_001277036.1	This study	N/A
p3xFLAG-CMV14-sheep ACE2, accession number: XP_011961657.1	This study	N/A
p3xFLAG-CMV14-tree shrews ACE2, accession number: QNV47311.1	This study	N/A
p3xFLAG-CMV14-R.affinis bat ACE2, accession number: QMQ39244	(Li et al., 2021)	N/A
p3xFLAG-CMV14-R.sinicus-YN bat ACE2, accession number: AGZ48803	(Li et al., 2021)	N/A
p3xFLAG-CMV14-R.sinicus-HB bat ACE2, accession number: ADN93475	(Li et al., 2021)	N/A
p3xFLAG-CMV14-R.macrotis bat ACE2, accession number: ADN93471.1	This study	N/A
p3xFLAG-CMV14-R.siamensis bat ACE2	This study	N/A
p3xFLAG-CMV14-R.pusillus bat ACE2, accession number: ADN93477.1	This study	N/A
p3xFLAG-CMV14-R.malayanus bat ACE2	This study	N/A
p3xFLAG-CMV14-R.shameli bat ACE2, accession number: UBB59645	This study	N/A
p3xFLAG-CMV14-R.pearsonii bat ACE2, accession number:QKE49996.1	This study	N/A
p3xFLAG-CMV14-R. luctus bat ACE2	This study	N/A
p3xFLAG-CMV14-R.ferrumequinum bat ACE2, accession number: XP_032963186.1	This study	N/A
p3xFLAG-CMV14-Hipposideros armiger bat ACE2, accession number: XP_019522954.1	This study	N/A
p3xFLAG-CMV14-H. pratti accession number: QKE49995.1	This study	N/A
p3xFLAG-CMV14-Pipistrellus abramus bat ACE2, accession number: ACT66266	This study	N/A
Software and algorithms		
Adobe Illustrator 2021	Adobe	https://www.adobe.com/
GraphPad Prism 9.3.1	GraphPad	https://www.graphpad.com/scientific-software/prism/
Image Lab 3.0	Bio-Rad	N/A
